# The Function of Non-invasive Prenatal Testing (NIPT) Request Forms in the Australian Context

**DOI:** 10.1007/s10728-025-00543-0

**Published:** 2025-10-24

**Authors:** Hilary Bowman-Smart, Molly Johnston, Michelle Taylor-Sands, Lisa Hui, Catherine Mills

**Affiliations:** 1 Australian Centre for Precision Health, University of South Australia, Adelaide, SA Australia; 2 Monash Bioethics Centre, Monash University, Melbourne, VIC Australia; 3 Murdoch Children’s Research Institute, Melbourne, VIC Australia; 4 Melbourne Law School, University of Melbourne, Melbourne, VIC Australia; 5 Department of Obstetrics and Gynaecology, Melbourne Medical School, The University of Melbourne, Melbourne, Australia; 6 Department of Obstetrics and Gynaecology, Mercy Hospital for Women, Mercy Health, Melbourne, Australia

**Keywords:** Prenatal screening, Non-invasive prenatal testing, Informed consent, Document analysis, Routinization, Genetics

## Abstract

Non-invasive prenatal testing (NIPT) is offered on a user-pays basis in Australia, with a range of providers and services available. A key concern raised about NIPT provision is the impact on informed consent of possible routinization of testing. Given these concerns, the documents, forms and informational material used in clinical practice are of critical importance. Test-specific request forms produced by commercial providers are frequently used in Australia, but there is a lack of clarity about the function or role of these forms. Some are labelled as “request forms”, while others include references to “informed consent”. Our aim in this study was to assess the range of functions that these forms may serve. We performed an online search for forms available from Australian providers in 2024 and applied a modified version of the Evaluative Linguistic Framework to a final list of eight forms. Our findings indicate significant heterogeneity and ambiguity in the function of these forms. We suggest these forms play a role in the performance of informed consent as a clinical ritual. Documents and forms can play a supportive role in counselling related to NIPT, but this supportive role should be recognized and articulated. These findings can inform the way healthcare professionals in Australia and other settings approach NIPT provision.

**Supplementary Information:**

The online version contains supplementary material available at https://doi.org/10.1007/s10728-025-00543-0.

## Introduction

Non-invasive prenatal testing (NIPT) has been widely adopted since its introduction in 2011 [1]. NIPT is usually offered from 10 weeks gestation, and can be used to screen for fetal conditions such as trisomy 21, or Down syndrome [2]. NIPT has higher performance compared to other prenatal screening methods such as combined first trimester screening (CFTS), and has seen rapid uptake in the prenatal care setting [3]. In tandem with developments in genomics, NIPT has the potential to provide a significant amount of detailed information about the fetus, such as microdeletions, single gene disorders, and polygenic scores [2].

Public funding for NIPT is also becoming more widespread across the world, with a range of approaches to implementation in countries such as the United Kingdom, France and the Netherlands [1, 3]. In countries where NIPT is not available through public healthcare systems or insurers, the cost is generally borne by patients. In Australia, NIPT is not publicly subsidized nor covered by private health insurance, and patients must pay approx. AUD$400–550 (USD $250–350) out of pocket. The Australian public healthcare system (“Medicare”) is a universal insurance scheme which provides set rebates for eligible care and treatment items listed on the Medicare Benefits Schedule (MBS). Clinicians and hospitals may charge patients a gap payment on top of the rebate, and additional services may be covered by private health insurers. While supporting broad access to healthcare services, this system can create inequities between patients who can afford non-subsidized tests and high gap payments, and those who cannot [4]. Older aneuploidy screening tests, such as CFTS, are covered by the MBS and thus are more affordable than NIPT [5].

Several commercial providers in Australia offer different brands of NIPT, including Harmony, Panorama, and *percept* [6]. Although NIPT is not publicly funded, uptake in Australia has been high, evidenced by a substantial drop in the number of Medicare item claims for prenatal diagnostic procedures and serum screening since the introduction of NIPT [5]. However, our recent empirical research has demonstrated that there is substantial variation in the provision of NIPT in clinical care in Australia, including healthcare professional knowledge, scope of test offered, and cost [7].

There is significant debate around information provision about NIPT in clinical practice, including how counselling should occur and how to understand informed choice and consent [8–11]. Prenatal screening programs are often centred around concepts such as reproductive autonomy and informed choice; according to Kuipers et al., ‘[t]he aim of prenatal screening is operationalized through informed consent’ [11]. However, with the increasing integration of genomics into prenatal testing, the limitations of informed consent as autonomy-promoting are becoming apparent [12]. Genomics can produce an enormous amount of information, such that it is not practically possible to communicate or make sense of all of it. This can result in “information overload”, where the sheer quantity of information can potentially frustrate informed decision-making rather than enhance it [13].

**Fig. 1:**
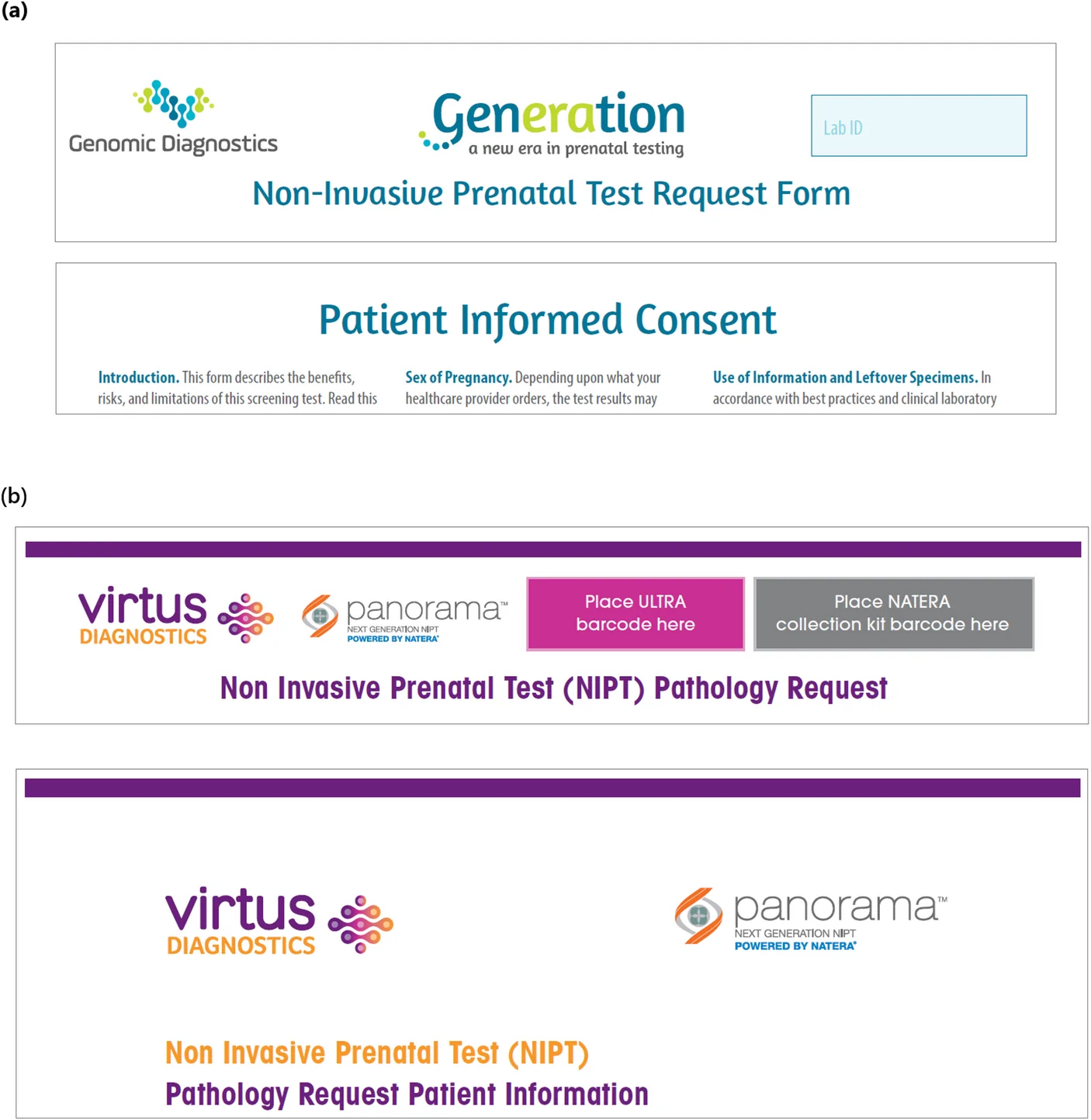
Variable labelling of NIPT forms. Headings of the first and second page of (**a)** NIPT-01 and (**b)** NIPT-07

Documents and forms can be used as part of information provision and counselling in clinical practice. In Australia, for patients to be able to access NIPT, clinicians generally need to fill out test-specific referral forms. Some of these forms are labelled as “request forms” and/or include references to “consent”; headings in others include “information” or are not explicitly labelled^1^ (see Fig. 1). They may be digital, or they may be printed out to become physical objects. Forms like these may play a significant role in the counselling, decision-making and consent processes for NIPT, but analyses of these documents are relatively sparse in the literature [14].

**Fig. 2:**
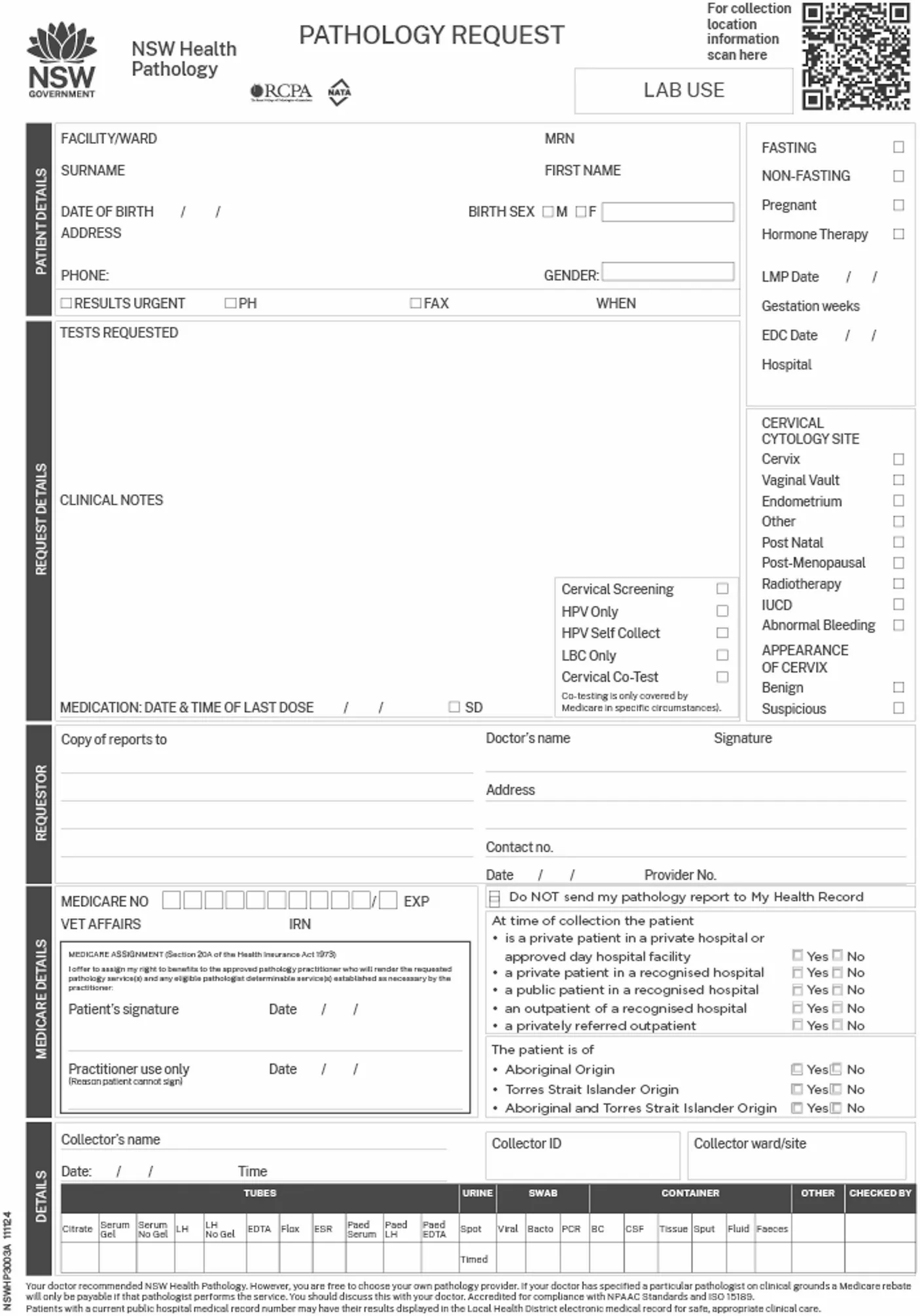
New South Wales (NSW) Health Pathology Request Form. This is an example of a generic request form for a state government pathology service, which may be used to request a range of tests or investigations, including antenatal. The patient signature is used for Medicare reimbursement, not as clinical consent for testing

**Fig. 3:**
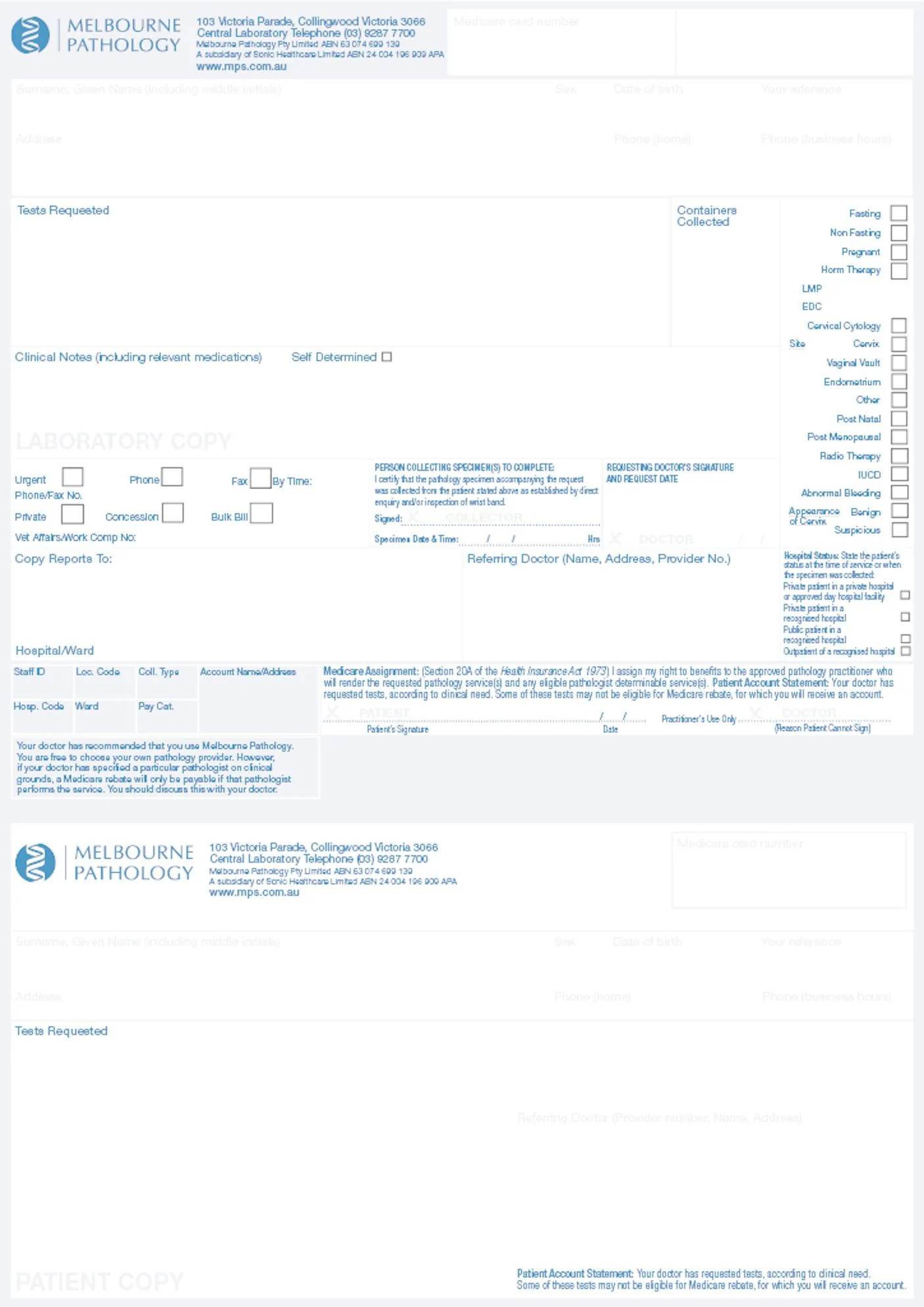
Melbourne Pathology Referral form. This is an example of a generic request form for a commercial pathology service. While this form carries the logo of a particular commercial pathology service, patients can take the form to other pathology providers if they prefer. Melbourne Pathology is part of Sonic Healthcare group, an Australian company which also offers an NIPT test through their “Sonic Genetics” service, using an NIPT-specific form (see Fig. 6)

In Australia, many commercial providers or services offering NIPT have developed their own branded version of these forms. These forms are used to request the provider’s own specific NIPT test. However, they differ significantly from standard referral or pathology request forms used in the Australian healthcare system. These standard or generic pathology request forms would be used to order other publicly subsidized antenatal investigations, such as ultrasounds, serum screening, or amniocentesis. These standard referrals or pathology requests generally do not include information about any specific test, references to consent, test branding, nor require patient signatures for consent (see Figs. 2 and 3).

This suggests that there may be some important differences in how the NIPT-specific forms function compared to generic pathology request forms. The NIPT forms frequently have features of pathology request forms, consent forms, informational leaflets, and/or marketing material. These documents may be presented as “consent forms” for patients choosing to use NIPT; they may function as simple administrative pathology request forms, sent directly to pathology services; they may be used for billing and financial records; or, they may be given to patients as informational material for decision-making. Thus, it is unclear what functions or roles these forms are performing within the context of the clinical encounter between patient and clinician.

The aim of this study is to examine the forms provided by major NIPT providers in Australia to understand the roles these NIPT-specific forms play in clinical practice. Previous studies have focused on analyzing NIPT informational material through the lens of quality assessment [14, 15]. However, here we are not assessing the *quality* of the forms in service of a particular function (e.g. assessing how well they function as “consent forms”); instead, we seek to understand what function(s) the forms are performing in the first place. By doing so, we can contribute to a better understanding of how NIPT is perceived to differ from other commonly performed investigations in antenatal care, and suggest underlying drivers for this difference. These findings may also be relevant to the provision of NIPT in other countries and regions, including where NIPT is being integrated into public healthcare systems.

## Methods

For our document analysis, we focused specifically on the forms rather than the associated information brochures. This is because the forms are required elements during the provision of NIPT and the clinical encounter, whereas the informational brochures are not.

### Search Strategy

To find the forms, an online search for publicly available documents was performed independently by two researchers (HBS and MJ). An initial search was performed in April 2021, and re-done in June 2024. The search engines Google and Bing were used. The first 10 pages of the search results were viewed. The search strings included the names of some specific tests known to the researchers, who have prior extensive knowledge of NIPT provision in Australia.

The queries used were: (Australia) AND (non-invasive prenatal testing OR NIPT OR non-invasive prenatal screening OR NIPS) [AND (percept OR harmony OR generation OR panorama OR nest OR GeneSyte or NIFTY)] AND (form OR consent form OR request form OR referral form). Websites were visited and links generated if there was a link to a form on that site.

Links were included in the initial search when they led to a downloadable form, in a format such as .pdf or .doc. Informational brochures and/or flyers were excluded. The computers used to perform the search had antivirus software which blocked access to some links.

These forms were downloaded and then the following inclusion criteria were applied to produce an initial list: (1) the forms indicated that they involved a test offered in Australia; (2) the forms were in English; (3) the forms were specific to the provision of NIPT (i.e. not other genetic or prenatal tests); (4) they were for use in the clinical/diagnostic setting; (5) the forms required a signature from a clinician and/or a patient.

Duplicates were then assessed using PDF comparison software. Exact duplicates were removed. Many forms were otherwise duplicates with different logos from different providers or services; in these cases, the most recent version of the forms with the first provider or service in alphabetical order was chosen.

The final selection of forms for analysis is not intended to be systematic or exhaustive, but rather to capture a reasonable scope and coverage of the forms used in the provision of key tests and/or larger providers or services in Australia.

### Analysis

For analysis of the forms, we drew on and adapted the Evaluative Linguistic Framework (ELF) developed by Clerehan et al. [16, 17]. We used this framework because of its focus on health communication and literacy, and its diverse applications. We did not want to use an analysis that presumed a specific function (e.g. evaluative criteria for consent forms [18]), nor a narrow focus on a particular element (e.g. readability [19])Knowing what, how and to whom the forms are communicating is critical to understanding the function in the clinical setting. The ELF has previously been used as a quality assessment framework for information pamphlets and consent forms for clinical trials, as well as assessment of comprehensibility of health questionnaires [20–22]. The ELF assesses nine elements of the document (Table 1): overall organizational or generic structure of the text; rhetorical elements; the writer-reader relationship; metadiscourse; signposts; technicality of vocabulary; lexical density; factual content of the text; and the format of the document.

**Table 1:** Components of the evaluative linguistic Framework, adapted from Clerehan et al. [16, 17]

Item	Description
Overall organizational or generic structure	The order of different sections or ‘moves’ in a text
Rhetorical elements	The function of each ‘move’ in relation to the reader e.g., to ‘instruct’
Writer - reader relationship	Nature of the relationship between the writer and the reader
Metadiscourse	Description of the purpose of the text
Headings	Signposts in the text
Technicality of vocabulary	The complexity of the terminology or language used in the text
Lexical density	The number of content words per clause, or proportion of content words out of all words. The higher the score, the denser the information is in a given portion of text.
Factual content of the text	Whether the information presented is accurate, evidence-based and up to date.^i^
Format	Visual aspects such as layout, font, and graphics

^i^ Factual content was assessed using a pre-determined list covering information that would be relevant to the concept of ‘informed consent’, informed from the Nuffield Council on Bioethics guidelines on non-invasive prenatal testing (2017) as well as feedback from clinicians and other professionals involved in the provision of NIPT - see Table 3

We built on the results from the ELF for further analysis, due to its focus on quality assessment, which was not the overarching purpose of our particular study. While that is relevant to our research question, our primary goal in this analysis is to examine the *function* of these forms (i.e. are they being *used as* consent forms? ) rather than their quality (i.e. are they *good* consent forms? ). For example, our examination of the “factual content of the text” here was not intended as a quality assessment of the forms *as* consent forms. Rather, this was to see if they contained the amount and type of information that suggested they are being *used* as consent forms.

## Results

The total number of documents with unique file names obtained through the search strategy was 87; the number of unique documents was 46, dated between 2014 and 2024. The final list of forms for analysis was generated as described above, resulting in 8 forms from 7 different Australian providers and services, dated between 2019 and 2023. Dates were ascertained from labelling within the document and/or the filename and/or file properties. The list of forms, and the forms themselves, can be found in the supplementary material.

The final set of forms included the following test brands and Australian providers: Generation (Abbott Pathology); Harmony (Australian Clinical Labs); non-branded NIPT offered by Sonic Genetics; Nest and Nest+ (Monash IVF); NIFTY (Genomics For Life); Panorama (Virtus Diagnostics); and percept (Victorian Clinical Genetics Services). This is not an exclusive list of providers or pathology services offering these tests in the Australian market, nor does it exclude the possibility that some providers, services or clinics may offer other NIPT brands and/or NIPT brands under another name.

In this manuscript, each form has been assigned an identifier (see supplementary material for forms).

### Overall Organizational or Generic Structure

**Fig. 4:**
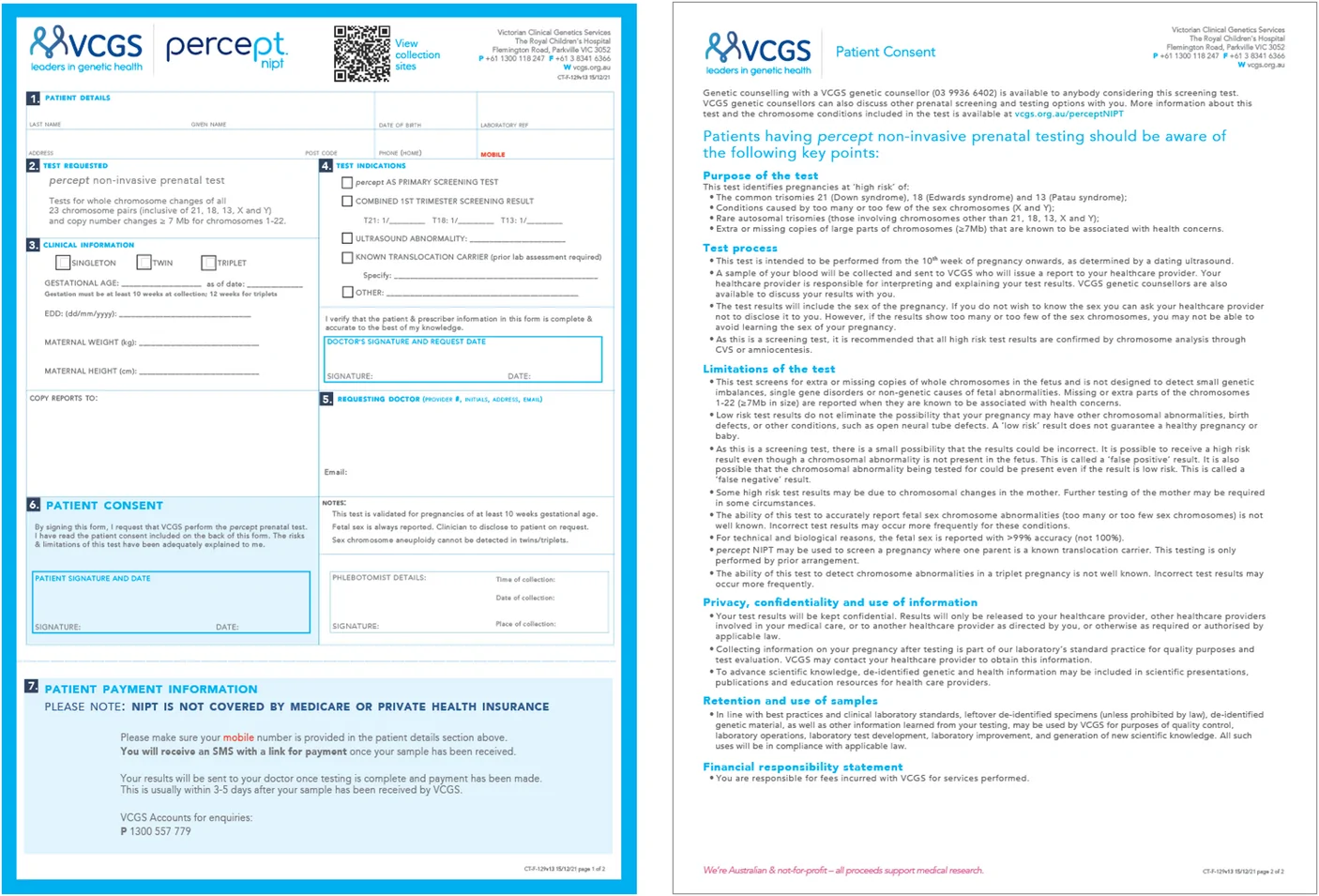
Example of two-page organisational structure (NIPT-08)

All the documents had two pages. For each document, the first page was the main form. The second page contained information: for 6/8, this was information about the test itself (e.g. scope and performance) and/or information about the test provider (see Fig. 4). For 2/8 – two test variants from the same provider, NIPT-04 and NIPT-05 – the second page largely contained maps and directions to clinic locations.

All the main forms (first pages) contained the following sections or ‘moves’^2^: information and/or branding for the test provider; request for patient information or details (the first fillable ‘move’ on all the forms); request for information about the requesting doctor; and clinical details. All forms had a move for describing the tests requested, with 7/8 (87.5%) offering a selection of different tests; NIPT-02 labelled this section as ‘*Test Menu Options*’.

The structure and sequence of moves was indicated visually through headings, boxes or columns. Many forms had boxes in two or three columns. One form (NIPT-08) numbered each box, which explicitly specifies the sequence in which the form should be read and filled out (see Fig. 4). For the other forms, however, the sequence of moves was somewhat ambiguous; a reader may go left to right skipping between columns, or down the leftward column(s) and then down the rightward column(s).

All forms had a move for a patient signature. Most forms (7/8) specified that this signature was required for patient “consent” or “informed consent”. One form (NIPT-03) included a reference to financial consent, labelling it as ‘*Patient and Financial Consent’* with a description of payment and refund processes. The one form without a reference to consent in this section (NIPT-07) did not explicitly specify the purpose of the patient signature but did state that the clinician signature was for ‘*Clinician Consent*’.

Many of these sections had accompanying statements that referred to the patient having read, or directing the patient to read, the other side of the form (4/8) or other documents containing ’*the information*’ about the test (2/7 - both NIPT-04 and NIPT-05). This has implications for the sequence of moves: it implies that you would first read the information side (and/or another document) and then return to the form. It is not clear whether this is how the sequence of moves would function in actual practice.

As digital documents, some forms had interactive components on the second page – e.g. NIPT-06 – to click “yes” or “no” for specific parts of the consent (e.g. incidental findings, use of unused test materials).

### Rhetorical Elements

In the first page of each document (i.e. the main form), the key function appeared to be ‘soliciting information’. The forms did this in a variety of ways. Most commonly, it was done visually with blank spaces, boxes or lines to write on (e.g. ‘*Name: ___________*).

A key related rhetorical element in the documents was ‘to instruct’. This occurs when the reader is explicitly told to do something (such as go to a particular place, or provide information). Examples include ‘*Select ONE [test name] test’* (NIPT-01), *‘(Please pay online)’* (NIPT-02), ‘*read informed consent section above*’ (NIPT-05 and NIPT-06), *’…please make an appointment by phoning [telephone number]’* (NIPT-05 and NIPT-06). Other instructions were implicit; ‘*Your results will be sent to your doctor once testing is complete and payment has been made*.’ (NIPT-08). This rhetorical function was also present on the second page of all the documents.

**Fig. 5:**
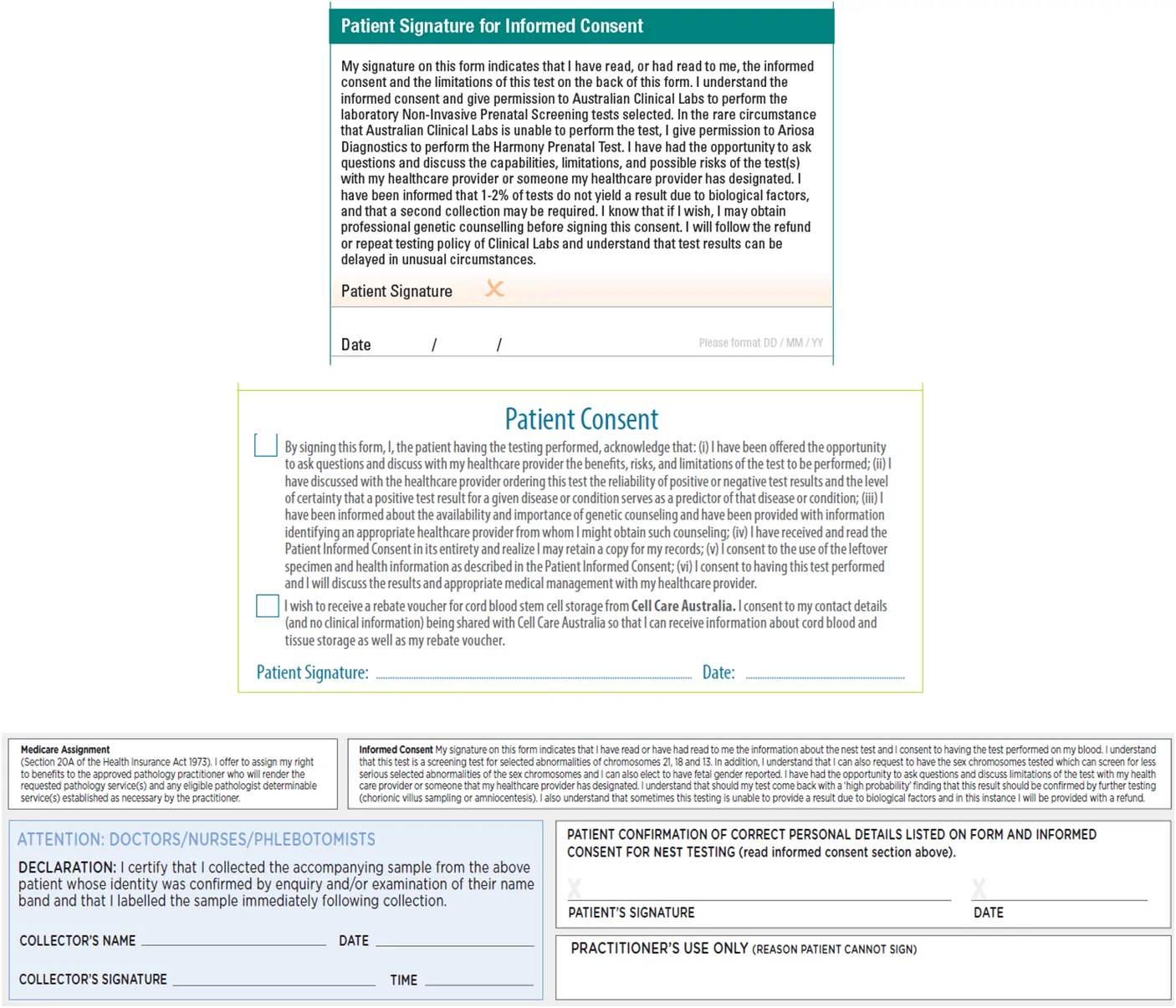
Examples of requests for patient signature with references to ‘consent’ or ‘informed consent’ (NIPT-02, NIPT-01, NIPT-04)

The sections that solicit a signature also served the function of ‘to certify’. Where the patient signature was requested, this generally served to confirm that the reader (the patient) has agreed to the test and/or has enough information to ‘consent’ (see Fig. 5). Where the clinician’s (and in some cases, the phlebotomist’s) signature was solicited, this served to confirm that they have engaged in certain required actions (e.g. counselling the patient). Signatures were usually indicating agreement to a statement in first person *(‘I understand…and give permission…’* (NIPT-02)), (‘*I confirm the patient has been counselled…*’ (NIPT-01)).

Other key rhetorical functions that were present throughout the documents were ‘to inform’ and ‘to explain’. There were instances of this in the main forms (e.g. ‘*Fetal sex is always reported’* (NIPT-08)). The second pages of the documents were generally devoted to this explanatory function, particularly where the second page was presented as information necessary for patient consent.

Some of the documents contained the rhetorical function of ‘to offer’ (e.g. *‘[Provider]…can refer you to a counselling service if you would like to avail yourself of this*’. (NIPT-01)). Some documents also contained the rhetorical function of ‘to direct elsewhere’, where the reader is told that further information or processes (e.g. payment) can be found in other documents and directed to these documents. This was generally done by providing URLs (which were directly clickable in the digital versions of the documents) and in three cases, a QR code.

### Writer-Reader Relationship

These documents generally had an ambiguous or alternating writer-reader relationship. The ‘writers’ of these documents were clearly positioned as the company that produces the test (and/or the pathology company). This positioning was generally achieved through branding and logos that identify the relevant compan(ies) or by using first person plural pronouns to position themselves as the writer of the text (e.g. ‘*We keep test results confidential*.’ (NIPT-01)).

However, while the writer was clear and consistent, the reader was not. These documents are designed to be read and interacted with by multiple readers - most notably, the patient and the clinician. There are also other readers beyond the patient and the clinician - for instance, all forms had a section that is explicitly addressed to those collecting and using the blood sample (e.g. nurse, phlebotomist, laboratory staff).

**Fig. 6:**
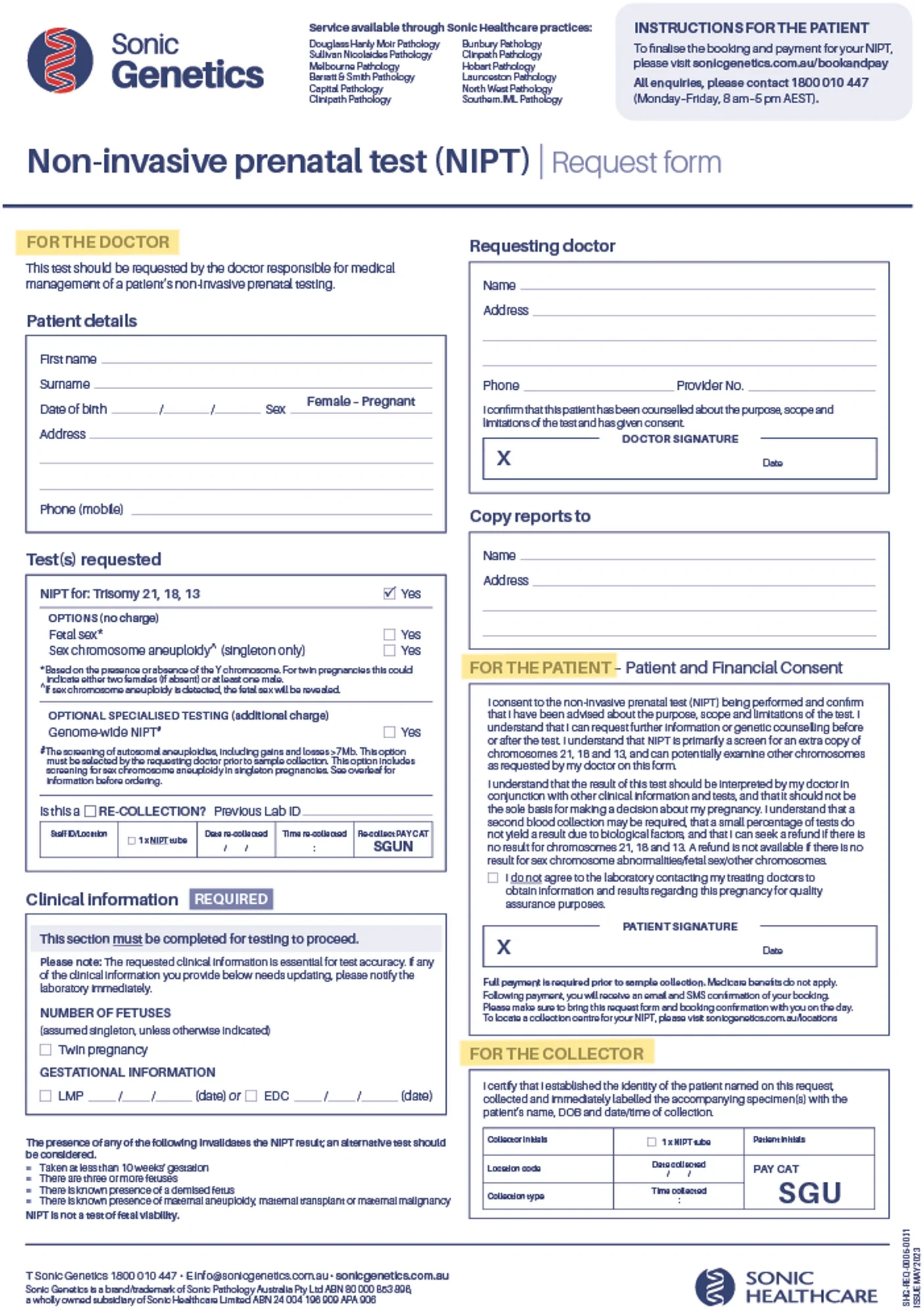
Form with explicit direction towards intended reader - see different sections with the headings “FOR THE DOCTOR”; “FOR THE PATIENT”; and “FOR THE COLLECTOR” (NIPT-03). Headings highlighted in yellow by authors

NIPT-03 explicitly labels who the reader of each section should be (*‘For the patient’, ‘For the doctor’, ‘For the collector’*) (see Fig. 6). For other forms, however, the reader must generally be inferred through context. For all documents, the patient was explicitly positioned as the key “reader” for some sections, particularly in sections referencing patient information or consent. For example, the ‘*Patient Consent*’ section in NIPT-01, which the patient must agree to with a signature, states *“…I have received and read the Patient Informed Consent in its entirety and realize I may retain a copy for my records…”.* Frequently, the clinician was positioned as the reader through reference to the “patient”. At points, this was very explicit: ‘*I attest that my patient…*’ (NIPT-02). One form (NIPT-07) included the request for patient signature in the same box as ‘Patient information’, which includes requests for personal details such as name and address, but also medical information about the pregnancy. Thus, while it is likely that the patient would fill this out, it is also possible that the clinician would.

The clinician was also positioned as the reader in some sections through technical language and initialisms, such as “CFTS” (Combined First Trimester Screening), “LMP” (Last Menstrual Period), “EDD” (Estimated Date of Delivery) (multiple forms). On the NIPT-04 and NIPT-05 forms, in the “essential information” section, they explicitly state that *‘patient may complete’* information about ethnicity and pregnancy history. The use of the modal verb “may” implies that there are multiple candidates for the position of reader, and it is up to the clinician - due to the use of the word “patient” - to decide who is best placed to interact with this section of the form.

### Metadiscourse

Metadiscourse was largely absent from the documents. On the NIPT-01 form, the second page of the document started with “This form describes the benefits, risks, and limitations of this screening test.”. The only metadiscourse in the other documents related to how the document is described in headings (discussed below).

### Signposts

Most of the documents (7/8 - all except for NIPT-08) had a main heading on the first page that describes the document as a “request” form. However, on the headings of the second page of the document, half of the documents included “consent” or “informed consent” while the other half refer to “patient information”, “information for patients”, or “information statement” (see Fig. 1).

Slightly over half of the forms (5/8) used color to differentiate the headings from the other text in the document. Other forms used a variety of methods to differentiate headings, including emphasis through bolding, capitalization, font size, and positioning.

Some forms had quite consistent approaches to subheadings. NIPT-08 has all subheadings in the same light blue color, size 6.3 font, with all letters capitalized and bolded (see Fig. 4). Other forms, such as the NIPT-05 and NIPT-06 forms, had subheadings in a variety of colors, font sizes, capitalization and formatting (some are underlined). On the second pages of the documents, 6/8 documents used subheadings to differentiate the information provided into sections.

### Technicality of Vocabulary

All the documents used some highly technical vocabulary and initialisms, though to varying degrees. These technical terms were frequently provided without clarification of their meaning. This included references to genetics terms (e.g., ‘*aneuploidies’*, ‘*translocation carrier’*, ‘*microdeletions’* etc.) and other medical terms, particularly relating to obstetrics, such as *’crown rump length’* and *’dizygotic’*. Furthermore, medical initialisms were used in 7/8 forms, such as *‘EDD’* (Estimated Due Date), ‘LMP’ (Last Menstrual Period), ‘MCMA’ (monochorionic monoamniotic), etc.

**Fig. 7:**
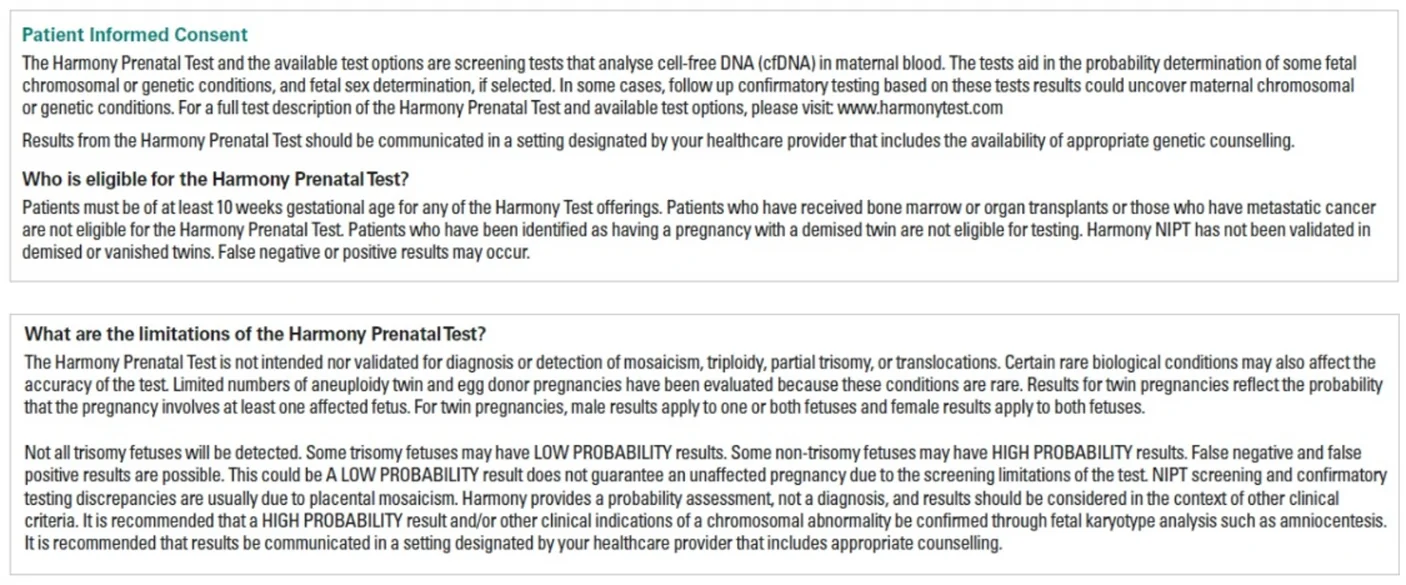
Complex and technical vocabulary on page with heading “Patient Informed Consent”. Selected sections from NIPT-02 include technical terms such as ‘aneuploidy’, ‘fetal karyotype’, ‘demised’ twin, and ‘translocations’

Some text that was directed to the patient-as-reader (indicated through headings and context) used this technical vocabulary. For example, under the heading of ‘*Patient Informed Consent*’, the NIPT-02 form stated that the test is ’…*not intended nor validated for diagnosis or detection of mosaicism*,* partial trisomy*,* or translocations...’* and that results may need to be confirmed through *‘fetal karyotype analysis such as amniocentesis’* (see Fig. 7).

The values expressed through the vocabulary are also relevant to the technicality of the vocabulary. Half (4/8) used neutral language such as *’probability’* or *’chance’* rather than ‘*risk’*, which carries negative connotations. Most of the forms (7/8)) referred to chromosome *’abnormalit(ies)*’. The NIPT-07 form also referred to chromosome ’*problems’*.

### Lexical Density

The lexical density was calculated by extracting all the text containing a clause and calculating the percentage of content words out of all words. A higher percentage indicates more complex and information-dense text. NIPT-04 had the lowest lexical density (47.48%) and NIPT-06 had the highest (56.03%) (Table 2).

**Table 2:** Lexical density of all eight documents

Form	Lexical density score
NIPT-01	53.9%
NIPT-02	53.06%
NIPT-03	50.92%
NIPT-04	47.48%
NIPT-05	48.28%
NIPT-06	56.03%
NIPT-07	50.74%
NIPT-08	52.51%

### Factual Content of Text

Here, as described in the methods, we adapted this component of the analysis and developed a list of information and content about NIPT that would be considered relevant to the decision-making of patients and prospective parent(s) (see Table 3). All forms outlined what conditions the test screens for; described NIPT as a screening test and that diagnostic testing would be required to confirm results; stated that NIPT can screen for fetal (chromosomal) sex; mentioned or referenced limitations for multiple pregnancies compared to singletons; avoided language implying reassurance or guarantees of a healthy pregnancy; and had an accompanying information brochure. Otherwise, inclusion of a range of important information (e.g. possibility of false positives or no results, variations in test performance for different conditions, incidental findings) was heterogeneous between forms.

**Table 3:** Analysis of the content of forms, including information presentation and framing

	Forms							
Content	NIPT-01	NIPT-02	NIPT-03	NIPT-04	NIPT-05	NIPT-06	NIPT-07	NIPT-08
1. Requests a patient signature for the purposes of consent	x	x	x	x	x	x		x
2. Outlines conditions NIPT screens for	x	x	x	x	x	x	x	x
3. Describes NIPT as a screening test	x	x	x	x	x	x	x	x
4. Explains explicitly what screening test means (i.e. that it is not diagnostic)		x	x			x		x
5. States possibility of false positives	x	x				x		x
6. States possibility of false negatives	x	x				x		x
7. Explains what a high-chance result means (i.e. that it is not diagnostic)		x					x	x
8. Explains what a low-chance result means (i.e. that it is not diagnostic)		x					x	x
9. Acknowledges possibility of difficult decision-making following NIPT								
10. States that diagnostic testing is required to confirm results	x	x	x	x	x	x	x	x
11. Outlines what diagnostic tests are available	x	x		x	x			x
12. Explains risk of diagnostic tests								
13. States diagnostic testing is voluntary								
14. Explains how NIPT works, as a test that analyses fetal genetic material in a maternal blood sample	x	x	x	x	x		x	x
15. Explains at what gestation NIPT can be done	x	x	x			x	x	x
16. States the cost of NIPT								
17. Turnaround time for NIPT results			x			x	x	
18. States accuracy of NIPT differs between screening targets		x	x			x		x
19. Provides information about the sensitivity or positive predictive value for each screening target								
20. States that NIPT can screen for fetal sex	x	x	x	x	x	x	x	x
21. Acknowledges information about fetal sex cannot be withheld if sex chromosome aneuploidies detected	x		x					x
22. Provides information on test performance or scope for singleton vs. multiple pregnancies	x	x	x	x	x	x	x	x
23. States NIPT does not screen for all genetic conditions	x	x	x			x	x	x
24. States that NIPT does not screen for all possible disabilities	x	x	x				x	x
25. Indicates the possibility of incidental findings	x	x				x		x
26. Explains how incidental findings would be returned						x		x
27. States the possibility of no result following NIPT		x	x	x	x	x	x	
28. Explains what happens if there is no result		x	x	x	x	x	x	
29. Explains how NIPT results are returned	x	x	x			x	x	x
30. Provides information on how long data will be stored								
31. Explains how data will be managed	x	x				x	x	x
32. Provides information on how long sample will be stored								x
33. Explains how sample will be managed	x	x				x	x	x
34. Recommends or indicates that there is an option of genetic counselling	x	x	x				x	x
35. Uses neutral language (i.e. “probability” or “chance” rather than “risk”)		x	x	x	x			
36. Does not use language indicating that NIPT can provide reassurance or guarantees (e.g. “peace of mind”)	x	x	x	x	x	x	x	x
37. Has accompanying information brochure available to download with form	x	x	x	x	x	x	x	x

### Format

The documents were all two pages. All documents were clearly branded visually with the use of logos at the top of the documents. Branded color schemes were also used (see Figs. 1, 4 and 6 for examples).

Within the forms, borders were frequently used to delineate sections, and the arrangement of ‘boxes’ suggests a particular sequence of moves. Most document were generally very visually consistent and/or clear, with consistent font sizes and styles. However, some forms (e.g. NIPT-04 and NIPT-05) had text that varied significantly throughout the document in both size and style. Some documents were quite visually cramped, with minimal space in between sections.

Although many of the forms used the word ‘consent’, generally, the text relating to the signature was in a very small font relative to other text (see Fig. 5). None of the forms had text relating to a signature for informed consent above a size 10 font.

## Discussion

This study reveals significant heterogeneity in the forms used to request NIPT in Australian clinical practice. The amount, quality, and accessibility of information varied significantly between forms. Many forms included references to consent but lacked features common or expected in clinical consent forms. Taken together, these variations suggest that the collective function of the forms is ambiguous, which has implications for counselling and informed consent.

### Ambiguity of Function

In many respects, the forms appeared to primarily function as pathology request forms. All forms requested relevant medical information from the clinician (e.g. maternal medical history, gestation, etc.). However, while much of the text was directed primarily at clinicians or phlebotomists collecting the blood sample, most of the forms also extensively positioned patients as the reader. This deviates from typical pathology request forms, which might only require a patient signature for Medicare billing purposes (see Figs. 2 and 3).

Another function of the forms is as marketing material. The use of visual markers such as branded colour schemes and logos serve to clearly identify what brand of NIPT is being ordered (see Figs. 1, 4 and 6). Across the world, many commercial providers of NIPT design and distribute collateral informational materials. These have been critiqued as biased or misleading, or functioning more as ‘marketing’ material to promote ‘consumer choice’ rather than serving to inform patients in a clinical context [23, 24]. Several forms in our study had a section for payment details, such that the document may also serve as a contract for sale. Given the relatively high cost of NIPT in Australia, the forms may also function as a form of financial disclosure of the out-of-pocket cost and/or consent for payment. This is explicitly stated in NIPT-03 which labels the section with the patient signature as “FOR THE PATIENT – Patient and Financial Consent”.

When NIPT is viewed as a commercial product, particularly if the process of payment occurs during the clinical encounter, the prospective parent(s) may be positioned as ’consumers’, with clinicians functioning to facilitate a commercial interaction. This dual positioning reflects concerns raised in the literature about how the use of language from commercial markets transforms the patient into consumer. The idea of patient-as-consumer can potentially serve to ‘empower’ patients to seek the type of care that they want, but may also position medical procedures as ‘products’ that can be purchased [25, 26]. This can in turn introduce facets such as advertising, marketing and commercial competition into the clinical encounter. Marketing and advertising for NIPT also occurs in non-clinical settings, with many patients seeking information from product websites, as well as discussions on social media websites such as Reddit and maternity forums [27, 28].

Furthermore, the nature of the choice(s) being made is itself ambiguous in this context. The concept of choice is particularly important in relation to prenatal testing and is central to the related concept of consent. As a medical procedure, there is choice on the part of the gestational parent about whether to provide a sample for NIPT. There is also a choice as to whether prospective parent(s) would prefer another type of prenatal test (e.g. combined first trimester screening, which is partially subsidized in the Australian healthcare system) or if they would like to proceed to invasive diagnostic testing such as amniocentesis. As a commercial product, there is also choice about what “brand” of test prospective parent(s) want to use, and what results they want to receive (e.g. information about fetal sex).

### Performance of (Informed) Consent

There is significant concern in the literature about how NIPT is provided within clinical care, the informational material(s) that are used, and (the potential lack of) informed consent when it comes to NIPT provision [8, 9, 11]. The critical importance of informed consent as a general concept or value is widely recognized in clinical practice, particularly where there is existing controversy about test routinization, as is the case with NIPT.

A function of these forms may be to facilitate and/or capture evidence of the patient providing informed consent. However, where these forms requested a patient signature for this purpose, the accompanying statement of information was in much smaller font than other text on the form. No form used font above a size 10, which is below the recommendation of at least size 12 for consent forms [29]. Small fonts can make critical information difficult to read and easy to overlook. Additionally, many of the forms used highly technical language (e.g. ‘aneuploidies’, ‘karyotype’, ‘exogenous’) and did not contain all the information considered necessary or important for consent in the context of NIPT (see Table 3).

Rather than functioning to obtain informed consent from prospective parent(s), we suggest that these documents actually function as *performances* of informed consent. By this we do not mean that clinicians or providers of NIPT in Australia are failing to obtain informed consent. Many clinical interactions are largely verbal, and a clinician may have explained information in appropriate detail to the patient; the patient may trust their clinician that the written contents of the form accurately reflects their conversation.

Our findings are, however, illustrative of how documents and informational material can prioritize the signal or ritual of consent over consent itself [30]. Many of these forms do not clearly indicate what patient consent is being sought for. One possibility is that they function straightforwardly as a consent form for a medical procedure: a signature is requested to signify informed consent to undergo NIPT. However, this practice deviates from other prenatal screening tests, both blood-based and ultrasound, where a signature is not usually required for clinical consent (see Figs. 2 and 3). Further, the organizational structure of the forms is at odds with a traditional consent form, where the signature would come after the relevant information. Almost all the information in the documents appears *after* the signature, on another page.

Although we did not aim to assess the quality of these documents as consent forms, their divergence from high-quality consent forms provides a guide as to how we can interpret their function. For example, the Nuffield Council states that informing patients about the possibility of false positives – a high probability result for a fetus without the condition – is essential information. However, only 4/8 of these forms stated this information (see Table 3) [31]. There are also many other features of these forms that do not adhere to guidelines for consent forms, such as technicality of vocabulary (as we noted, highly technical language was frequently used) as well as how additional information about the test was visually presented (if at all).

Given concerns around informed consent and routinization in relation to NIPT, a patient signature may also be requested in response to questions of liability (legal, social or financial) and to maintain the reputation of the provider. Requesting the patient’s signature may be used as evidence that the providers have performed their ‘due diligence’ in ensuring informed consent, both with regards to the medical aspects of the test as well as the financial cost. The patient signature can be used to fend off potential critiques of ‘routinization’, because it explicitly indicates that the patient has actively been involved in the decision-making. A comparison can be drawn to the process of reading privacy policies and ‘terms of service’ documents. Research suggests that the majority of people do not read such terms or policies at all before agreeing, and if they do, they only briefly skim them [32]. Thus, the documents serve to “operationalize” consent and can function as potential proof to a third party that certain processes or procedures have been followed [33].

In addition to clinical consent, there may be other reasons a patient signature for ‘consent’ is required. One example may be the retention of data. Several forms where the patient indicated their consent through a signature included statements on the second page about the storage and use of either the data or the physical samples. It may be that companies are also interested in the ability to continue to use samples or data for research purposes. The company BGI Group produces the NIFTY NIPT test, available in more than 50 countries including Australia. They have faced scrutiny regarding possible secondary use of data collected through NIPT, including concerns that data may be shared with government bodies; this was strongly denied by BGI [34]. The secondary use of data raises concerns around privacy, security, and the right to withdraw [35, 36]. Using clinical data for research purposes creates further complexities, given that some people may not realize or understand the full extent to which their data could be used. While these debates are not new, how these issues apply to genetic and genomic data collected through prenatal testing warrants further consideration, especially since samples collected for NIPT can capture genetic information about both the fetus (a future person) and the pregnant person.

This highlights an ongoing gap between the theory and practice of informed consent in prenatal screening, with informed consent for procedures such as ultrasound and serum screening being “completely abandoned…[or]…largely neglected.” [12]. The performance of informed consent that we have described here situates the current clinical practice of NIPT in a sort of middle ground. A certain conception of informed consent may be considered important in the abstract, but for various reasons it differs from what is applied in practice. Formalized or structured consent processes have elsewhere been described as a form of “clinical ritual of trust”, which aims not to truly obtain informed consent, but rather to establish trust and provide reassurance that a patient has not been deceived or coerced [37]. Such rituals can have important functions in the formation, conduct and dynamics of relationships in clinical care [30]. The ambiguity of the specific function of these documents that we have identified here (e.g. consent, pathology request, marketing) may be in part because they are subordinate to this overarching function in a clinical ritual.

From an anthropological perspective, an emphasis on forms, written materials, and signatures – despite recognition of their insufficiency or limitations - may also illustrate how the document as object can take on the qualities of a fetish (an object perceived to have certain powers) [38]. This emphasis on forms also reflects a legalistic approach to medical decision-making which Kuczewski describes as “an event model of informed consent”, as opposed to consent as an ongoing or dynamic process [39]. From this perspective, the signing of a form is seen as constituting the ‘event’ of receiving legally valid consent from the patient and/or discharging the clinician’s duty to inform. Although it is not necessary for consent in the clinical setting to be given in writing (other methods such as verbal consent are also generally satisfactory), doing so creates a clear record or provides evidence that consent has been given.

Despite several of the forms being presented as part of patient ‘consent’, their structure and content are at odds with this function. We recognise that important information pertaining to NIPT may have been discussed within the clinician-patient interaction and/or provided through other informational material, and informed consent may nevertheless have been obtained. Even so, we suggest the positioning of the documents as ‘consent’ forms is a secondary function and is not necessarily prioritized (either in intent or in practice) as the primary function of the documents. Thus, the use of these forms may serve as a performance of informed consent, rather than as part of a meaningful process.

Our findings do not necessarily mean that further development of written consent materials in prenatal care should be prioritized as the best means of addressing some of the issues we have discussed. Written material may be useful as a means of structuring dialogue and as reference material, but is not sufficient by itself in obtaining informed consent and promoting reproductive autonomy [33, 40]. The three-step counselling model from Kater-Kuipers et al. (2020) places an exploration of the prospective parent(s)’ personal values as the first step [11]. The type of forms currently in use in NIPT provision could still play an important supportive role in the counselling and decision-making process. However, where the forms play such a role, it is important to explicitly recognize and articulate this supportive role, rather than present the forms as primarily serving functions they do not.

### Limitations

A key limitation of this study is that we are examining the functions of these forms through the static presentation of a blank document. The very nature of forms as interactive documents – particularly where multiple actors are likely to “interact” with them, as is the case here – suggests the need for a method more suited to the analysis of this type of document. Furthermore, our study does not provide insight into how these forms may be used in actual practice within the context of the clinician-patient interaction, as well as what and how other types of information or documents may be provided to the patient.

To best understand the function of these forms within the clinician-patient interaction, further research that can directly observe how they are used in the clinical setting would be beneficial. For example, that could include a more extensive ethnographic study. This would help answer other questions that illuminate the way prospective parent(s) interact with and understand NIPT in a commercial context – for example, whether patients are bringing the forms to the doctors, or if the doctors are providing the forms themselves. A further key question is whether clinicians are providing a choice of different providers, or only one (and thus one type of form). Approximately 60% of respondents to our recent survey of Australian healthcare providers indicated that they refer patients to one particular brand of NIPT, with test performance and convenience of blood collection being key reasons cited for brand choice [7]. Another key limitation of our study is that some of these forms are specific to the Australian context. Some forms did use American spelling (e.g. “realize” vs. “realise”, see Fig. 5), suggesting that some features or sections may be directly copied from documents used in the United States. Nevertheless, these results may provide insights for other contexts – particularly those where NIPT is still commercially provided – and further research in those contexts will be useful.

## Conclusion

In this study we aimed to analyze the function of the forms that accompany the provision of NIPT in the Australian context, where NIPT is provided on a commercial, user-pays basis. This is important because of significant concerns about how NIPT is offered, described, framed, consented to and even “sold” within the context of the clinical encounter. We found that there is significant ambiguity of function in how NIPT forms are designed and presented. All the forms we examined have elements that suggest they serve as pathology request forms. The format of some forms, particularly the use of visual and linguistic markers such as logos and branding, framed them as a sort of commercial marketing material. Notably, many forms used language or elements (e.g. signatures) that indicate a function of patient consent. However, our analysis indicates that the function of substantively obtaining truly informed consent is de-prioritised at best, and the forms function more as part of a performance of informed consent.

## Supplementary Information

Below is the link to the electronic supplementary material.

Supplementary Material 1

Supplementary Material 2

## Data Availability

All data are publicly available; please see supplementary material.

## Footnotes

1 From here on we will refer to these forms as “the forms”.

2 ‘Moves’ are the different components of the document.

## References

[1] Ravitsky, V., Roy, M-C., Haidar, H., Henneman, L., Marshall, J., Newson, A. J., et al. (2021). The emergence and global spread of noninvasive prenatal testing. *Annual Review of Genomics and Human Genetics*, *22*, 309–338. 10.1146/annurev-genom-083118-015053 33848430

[2] Hui, L., Bianchi, D. W., Noninvasive Prenatal, D. N. A., & Testing (2017). The vanguard of genomic medicine. *Annual Review of Medicine*, *68*, 459–472. 10.1146/annurev-med-072115-033220 PMC10066603 27732785

[3] Sebire, E., Rodrigo, C. H., Bhattacharya, S., Black, M., Wood, R., & Vieira, R. (2024). The implementation and impact of non-invasive prenatal testing (NIPT) for down’s syndrome into antenatal screening programmes: A systematic review and meta-analysis. *PLOS ONE*, *19*, e0298643. 10.1371/journal.pone.0298643 PMC11098470 38753891

[4] Fox, H., Topp, S. M., Callander, E., & Lindsay, D. (2019). A review of the impact of financing mechanisms on maternal health care in Australia. *Bmc Public Health*, *19*, 1540. 10.1186/s12889-019-7850-6 PMC6873587 31752792

[5] Hui, L., & Halliday, J. A decade of non-invasive prenatal screening in australia: National impact on prenatal screening and diagnostic testing. *Australian and New Zealand Journal of Obstetrics and Gynaecology* 2023;n/a. 10.1111/ajo.13638 PMC10952159 36529142

[6] Kluckow, E., Halliday, J., Poulton, A., Lindquist, A., Hutchinson, B., Bethune, M., et al. (2019). Association between timing of diagnosis of trisomy 21, 18, and 13 and maternal socio-economic status in Victoria, australia: A population‐based cohort study from 2015 to 2016. *Prenatal Diagnosis*, *39*, 1254–1261. 10.1002/pd.5577 31691307

[7] Johnston, M., Hui, L., Bowman-Smart, H., Taylor-Sands, M., Pertile, M. D., & Mills, C. (2024). Disparities in integrating non-invasive prenatal testing into antenatal healthcare in australia: A survey of healthcare professionals. *Bmc Pregnancy and Childbirth*, *24*, 355. 10.1186/s12884-024-06565-1 PMC11092221 38745131

[8] Dane, A. C., Peterson, M., & Miller, Y. D. (2018). Talking points: Women’s information needs for informed decision-making about noninvasive prenatal testing for down syndrome. *Journal of Genetic Counseling*, *27*, 1258–1264. 10.1007/s10897-018-0250-8 29550971

[9] Horn, R. (2022). NIPT and the concerns regarding ‘routinisation’. *European Journal of Human Genetics*, 1–2. 10.1038/s41431-022-01053-6 PMC9177732 35132175

[10] Montgomery, S., & Thayer, Z. M. (2020). The influence of experiential knowledge and societal perceptions on decision-making regarding non-invasive prenatal testing (NIPT). *Bmc Pregnancy and Childbirth*, *20*, 630. 10.1186/s12884-020-03203-4 PMC7574418 33076858

[11] Kater-Kuipers, A., de Beaufort, I. D., Galjaard, R-J-H., & Bunnik, E. M. (2020). Rethinking counselling in prenatal screening: An ethical analysis of informed consent in the context of non-invasive prenatal testing (NIPT). *Bioethics*, *34*, 671–678. 10.1111/bioe.12760 PMC7586798 32621525

[12] Ravitsky, V. (2017). The shifting landscape of prenatal testing: Between reproductive autonomy and public health. *Hastings Center Report*, *47*(Suppl 3), S34–40. 10.1002/hast.793 29171889

[13] Horn, R., & Parker, M. (2018). Opening pandora’s box? Ethical issues in prenatal whole genome and exome sequencing. *Prenatal Diagnosis*, *38*, 20–25. 10.1002/pd.5114 PMC5836985 28695688

[14] Michie, M., Kraft, S. A., Minear, M. A., Ryan, R. R., & Allyse, M. A. (2016). Informed decision-making about prenatal CfDNA screening: An assessment of written materials. *Ethics Medicine and Public Health*, *2*, 362–371. 10.1016/j.jemep.2016.05.004 PMC5044756 27699200

[15] Daley, R., Hill, M., & Lewis, C. (2017). Evaluation of patient information leaflets for non-invasive prenatal testing for down’s syndrome. *British Journal of Midwifery*, *25*, 585–592. 10.12968/bjom.2017.25.9.585

[16] Clerehan, R., Guillemin, F., Epstein, J., & Buchbinder, R. (2016). Using the evaluative linguistic framework for questionnaires to assess comprehensibility of Self-Report health questionnaires. *Value in Health*, *19*, 335–342. 10.1016/j.jval.2016.01.008 27325325

[17] Clerehan, R., Buchbinder, R., & Moodie, J. (2005). A linguistic framework for assessing the quality of written patient information: Its use in assessing methotrexate information for rheumatoid arthritis. *Health Education Research*, *20*, 334–344. 10.1093/her/cyg123 15494359

[18] Spatz, E. S., Suter, L. G., George, E., Perez, M., Curry, L., Desai, V., et al. (2020). An instrument for assessing the quality of informed consent documents for elective procedures: Development and testing. *British Medical Journal Open*, *10*, e033297. 10.1136/bmjopen-2019-033297 PMC7247404 32434933

[19] Daraz, L., Morrow, A. S., Ponce, O. J., Farah, W., Katabi, A., Majzoub, A., et al. (2018). Readability of online health information: A Meta-Narrative systematic review. *American Journal of Medical Quality*, *33*, 487–492. 10.1177/1062860617751639 29345143

[20] Sand, K., Eik-Nes, N. L., & Loge, J. H. (2012). Readability of informed consent documents (1987–2007) for clinical trials: A linguistic analysis. *Journal of Empirical Research on Human Research Ethics*, *7*, 67–78. 10.1525/jer.2012.7.4.67 23086049

[21] Petkovic, J., Epstein, J., Buchbinder, R., Welch, V., Rader, T., Lyddiatt, A., et al. (2015). Toward ensuring health equity: Readability and cultural equivalence of OMERACT Patient-reported outcome measures. *Journal of Rheumatology*, *42*, 2448–2459. 10.3899/jrheum.141168 PMC4765994 26077410

[22] Morony, S., Webster, A. C., Buchbinder, R., Kirkendall, S., McCaffery, K. J., & Clerehan, R. (2018). A linguistic analysis of health literacy demands of chronic kidney disease patient education materials. *Health Lit Res Pract*, *2*, e1–14. 10.3928/24748307-20171227-01 PMC6608909 31294272

[23] Holloway, K., Simms, N., Hayeems, R. Z., & Miller, F. A. (2022). The market in noninvasive prenatal tests and the message to consumers: Exploring responsibility. *Hastings Center Report*, *52*, 49–57. 10.1002/hast.1329 35099072

[24] Farrell, R. M., Agatisa, P. K., Mercer, M., & Coleridge, M. B. (2015). Online direct-to-consumer messages about non-invasive prenatal genetic testing. *Reproductive Biomedicine & Society Online*, *1*, 88–97. 10.1016/j.rbms.2016.02.002 PMC6001346 29911190

[25] Plastow, M. (2010). The intrusion of the discourse of economics into the clinical space I: From patient to consumer. *Australas Psychiatry*, *18*, 298–302. 10.3109/10398561003731171 20645893

[26] Goldstein, M. M., & Bowers, D. G. (2015). The patient as consumer: Empowerment or commodification? *Journal of Law Medicine & Ethics*, *43*, 162–165. 10.1111/jlme.12203 25846046

[27] Marcon, A. R., Ravitsky, V., & Caulfield, T. (2021). Discussing non-invasive prenatal testing on reddit: The benefits, the concerns, and the comradery. *Prenatal Diagnosis*, *41*, 100–110. 10.1002/pd.5841 33058217

[28] CrabbeRES, Stone, P., & Filoche, S. K. (2019). What are women saying about non-invasive prenatal testing? An analysis of online pregnancy discussion forums. *Prenatal Diagnosis*, *0*. 10.1002/pd.5500 31172546

[29] Coleman, E., O’Sullivan, L., Crowley, R., Hanbidge, M., Driver, S., Kroll, T., et al. (2021). Preparing accessible and understandable clinical research participant information leaflets and consent forms: A set of guidelines from an expert consensus conference. *Research Involvement and Engagement*, *7*, 31. 10.1186/s40900-021-00265-2 PMC8130271 34006326

[30] Arnold, M. H., Komesaroff, P., & Kerridge, I. (2020). Understanding the ethical implications of the rituals of medicine. *Internal Medicine Journal*, *50*, 1123–1131. 10.1111/imj.14990 32929818

[31] Nuffield Council on Bioethics (2017). Non-invasive prenatal testing: ethical issues.

[32] Obar, J. A., & Oeldorf-Hirsch, A. (2020). The biggest lie on the internet: Ignoring the privacy policies and terms of service policies of social networking services. *Information Communication & Society*, *23*, 128–147. 10.1080/1369118X.2018.1486870

[33] Xu, A., Baysari, M. T., Stocker, S. L., Leow, L. J., Day, R. O., & Carland, J. E. (2020). Researchers’ views on, and experiences with, the requirement to obtain informed consent in research involving human participants: A qualitative study. *BMC Medical Ethics*, *21*, 93. 10.1186/s12910-020-00538-7 PMC7531157 33008387

[34] Needham, K., & Baldwin, C. (2021). China’s gene giant harvests data from millions of pregnant women. Reuters.

[35] Kaye, J. (2012). The tension between data sharing and the protection of privacy in genomics research. *Annu Rev Genomics Hum Genet*, *13*, 415–431. 10.1146/annurev-genom-082410-101454 PMC4337968 22404490

[36] Mittelstadt, B. D., & Floridi, L. (2016). The ethics of big data: Current and foreseeable issues in biomedical contexts. *Science and Engineering Ethics*, *22*, 303–341. 10.1007/s11948-015-9652-2 26002496

[37] O’Neill, O. (2003). Some limits of informed consent. *Journal of Medical Ethics*, *29*, 4–7. 10.1136/jme.29.1.4 PMC1733683 12569185

[38] Wynn, L., & Israel, M. (2018). The fetishes of consent: Signatures, paper, and writing in research ethics review. *American Anthropologist*, *120*, 795–806. 10.1111/aman.13148

[39] Kuczewski, M. G. (1996). Reconceiving the family: The process of consent in medical decisionmaking. *Hastings Center Report*, *26*, 30–37. 10.2307/3528574 8722524

[40] Lühnen, J., Mühlhauser, I., & Steckelberg, A. (2018). The quality of informed consent Forms—a systematic review and critical analysis. *Dtsch Arztebl Int*, *115*, 377–383. 10.3238/arztebl.2018.0377 PMC6039714 29932049

