# Supplementary material for: The Function of Non-invasive Prenatal Testing (NIPT) Request Forms in the Australian Context
Source: Health Care Anal. 2025 Oct 24;34(3):783–807. doi: 10.1007/s10728-025-00543-0 (PMC13675290; doi:10.1007/s10728-025-00543-0)
Supplement: Supplementary file 1 [file 10728_2025_543_MOESM1_ESM.pdf]

Genomic Diagnostics

Generation

a new era in prenatal testing

Lab ID

Non-Invasive Prenatal Test Request Form

Patient Information

Surname: ....

First Name: ....

DOB: D D M M Y Y Y Y Phone: ....

Address: ....

.... Postcode: ....

Email: ....

Patient Consent

☐ By signing this form, I, the patient having the testing performed, acknowledge that: (i) I have been offered the opportunity to ask questions and discuss with my healthcare provider the benefits, risks, and limitations of the test to be performed; (ii) I have discussed with the healthcare provider ordering this test the reliability of positive or negative test results and the level of certainty that a positive test result for a given disease or condition serves as a predictor of that disease or condition; (iii) I have been informed about the availability and importance of genetic counseling and have been provided with information identifying an appropriate healthcare provider from whom I might obtain such counseling; (iv) I have received and read the Patient Informed Consent in its entirety and realize I may retain a copy for my records; (v) I consent to the use of the leftover specimen and health information as described in the Patient Informed Consent; (vi) I consent to having this test performed and I will discuss the results and appropriate medical management with my healthcare provider.

☐ I wish to receive a rebate voucher for cord blood stem cell storage from **Cell Care Australia.** I consent to my contact details (and no clinical information) being shared with Cell Care Australia so that I can receive information about cord blood and tissue storage as well as my rebate voucher.

Patient Signature: .... Date: ....

Requesting Doctor to Sign

Name: ....

Address: ....

.... Postcode: ....

Phone: .... Fax: ....

Dr Provider No. ....

I confirm the patient has been counselled on the purpose, scope and limitations of the test and has given consent.

Signature: ....

Report Copy Doctor

Name: ....

Address: ....

.... Postcode: ....

Phone: .... Fax: ....

NIPT-01

Test Requested

Step 1: Select **ONE** Generation test:

☐ **Generation** — 1 x Streck Tube
- T21, T18, T13 & specific sex chromosome aneuploidies

Sex Chromosome aneuploidies is not available for twins

☐ **Generation 46** — 1 x Streck Tube
- Chromosomal and subchromosomal aneuploidies of chromosomes 1-22 & specific sex chromosome aneuploidies

Sex Chromosome aneuploidies is not available for twins

☐ **Generation Plus** — 2 x Streck Tubes
- T21, T18, T13 & specific sex chromosome aneuploidies
- Microdeletions (See overleaf)

Generation Plus is not available for twins

Collection available Monday ONLY

Regional WA: collection Mon AM ONLY

Step 2: Indicate whether fetal sex is required:

I want fetal sex results included in this report ☐ **YES** ☐ **NO**

If you do not tick one of the boxes above fetal sex results WILL automatically be included in the report. *Fetal sex identification is not available for twins. A high-risk chromosome aneuploidy result will be reported when the fetal sex identification is not requested.*

Test Indications

Choose at least one:

☐ IVF Pregnancy

☐ Recurrent miscarriage/pregnancy loss

☐ Advanced Maternal Age

☐ Positive Serum Screen /High risk CFTS

☐ Abnormal Ultrasound

☐ Hx suggestive of increased risk for the specified chromosome aneuploidies

☐ Patient Request

☐ Other (specify in Clinical Details)

Clinical Details

EDD (mandatory): D D M M Y Y

No. of fetuses: ☐ Singleton ☐ Twin

Comments: ....

This prenatal test is validated for singleton and twin pregnancies with gestational age of at least 10 weeks 0 days, as estimated by last menstrual period, crown rump length, or other appropriate method (equivalent to 8 weeks fetal age as determined by date of conception).

Do not send reports to My Health Record ☐

DATA ENTRY INSTRUCTIONS: **PANEL CODE:** **Generation** NIP **Generation 46** NAA **Generation Plus** NPX

QR Pay:

Payment Information - This test is NOT covered by Medicare or Private Health Funds.

- Full payment is required prior to blood collection
- Go to **generationnipt.com.au** to pay and locate a Generation collection centre
- Call 1800 822 999 (Mon-Fri, 8am-6pm AEST) for enquiries or assistance

Receipt Number:

PERSON COLLECTING SPECIMEN TO COMPLETE:

I certify I established the identity of the patient named on this request, collected and immediately labelled the accompanying specimen with the patient's details.

Initials: .... ACC Code / Location: .... Date of draw: D D M M Y Y Time: __ __ : __ __ am / pm

GD-RF003_AV11_May23

Patient Informed Consent

Introduction. This form describes the benefits, risks, and limitations of this screening test. Read this form carefully before making your decision about testing. It is recommended that you seek genetic counselling prior to undergoing this testing. More information regarding this test is available at www.genomicdiagnostics.com.au

Purpose. The purpose of this tests is to screen your pregnancy for chromosomal abnormalities, that cause too many or too few copies of all or part of a chromosome. Depending on the Generation test chosen, screening includes common trisomies 21, 18 and 13, with options for screening for all autosomes (chromosomes 1 to 22), sex chromosomes and microdeletions.

Your options. There are three Generation tests available. Generation screens for the common trisomies (chromosomes 21, 18 & 13), as well as specific sex aneuploidies.
Generation Plus includes these plus 5 microdeletion syndromes - 22q11 deletion (DiGeorge), 15q11 deletion (Angelman/Prader-Willi), 1p36 deletion, 4p- (Wolf-Hirschhorn) & 5p- (Cri-du-chat). Generation 46 screens for chromosomal and subchromosomal aneuploidies of chromosomes 1-22 & specific sex chromosome aneuploidies.

Test Procedure. Payment for your NIPT is required prior to having your blood collected. Collection centre locations are available online when you pay for your test. No booking is required. A tube of your blood will be drawn at your most convenient centre and sent to Genomic Diagnostics, a wholly Australian owned and operated business, where it will be analysed and the results sent back to your doctor. Generation Plus tests are analysed at an accredited laboratory in California, USA.

How this Test Works. This test is not intended to be performed prior to the 10th week of pregnancy, as estimated by last menstrual period, crown rump length, or other appropriate method. The test screens for specific chromosomal abnormalities by looking at the DNA (genetic material) in your blood. To determine whether too few or too many chromosomes are present, this test uses a technology called 'massively parallel DNA sequencing' to count the number of copies of the specific chromosomes, and then uses a proprietary method to determine if there are too many or too few copies of the chromosomes in your pregnancy.

Sex of Pregnancy. Depending upon what your healthcare provider orders, the test results may include the sex of the pregnancy. If you do not wish to know the sex, please tell your healthcare provider to indicate this by ticking the appropriate box under Fetal Sex identification in the Test Menu Options section of the request form. Depending upon the test ordered you may not be able to prevent learning the sex of your pregnancy. In rare instances (<1%), incorrect fetal sex results can occur due to either technical or biological reasons.

Limitations of the Test. This is a screening test that only looks for the specific chromosomal abnormalities tested for. This means other untested chromosomal abnormalities may be present and could cause health concerns. This test does not test the health of the mother, although in some rare cases some high risk test results may be due to chromosomal changes in the mother and may require further investigation. If definitive diagnosis is desired, chorionic villous sampling or amniocentesis should be considered. Normal test results do not eliminate the possibility that your pregnancy may have other chromosomal abnormalities, birth defects, or other conditions, such as open neural tube defects. In addition, a normal result does not guarantee a healthy pregnancy or baby. This test, like many screening tests, has limitations including false positive and false negative rates. This means that the chromosomal abnormality being tested for may be present even if you receive a negative result (this is called a "false negative"); Or that you may receive a positive result for the chromosomal abnormality being tested for, even though it was not really present (this is called a "false positive"). For these reasons it is strongly recommended that no irreversible clinical decisions be made based on these screening results alone. Further testing of the pregnancy and in some cases you, may be needed to confirm your test results which could result in additional expense to you and additional invasive testing procedures (e.g., amniocentesis or chorionic villus samples).

Privacy. We keep test results confidential. Your test results will only be released to your requesting healthcare provider or to another healthcare provider as directed by you, or otherwise as required or authorised by applicable law.

Use of Information and Leftover Specimens. In accordance with best practices and clinical laboratory standards, leftover de-identified specimens (unless prohibited by law) as well de-identified genetic and other information learned from your testing may be used by Genomic Diagnostics for purposes of quality control, laboratory operations, laboratory test development, and laboratory improvement.
All such uses will be in compliance with applicable law. Collection of information regarding your pregnancy after testing is part of a laboratory's standard practice for quality purposes. Genomic Diagnostics may contact your health provider to obtain this information.

Test Results. Your test results will be sent to the healthcare provider that ordered the test. Speak with them if you would like a copy of the test results. Your healthcare provider is responsible for interpreting the test results and explaining the meaning to you. Genomic Diagnostics does not provide genetic counselling services directly to patients but can refer you to a counselling service if you would like to avail yourself of this.

Contact Details. By ticking the box under Patient Consent, I agree to receive a cord blood stem cell storage rebate voucher valued up to $250 from Cell Care Australia. I consent to my contact details (but no clinical information) being shared with Cell Care Australia so that I can receive information about cord blood and tissue storage as well as my rebate voucher.

NIPT-01

Genomic Diagnostics

Generation
a new era in prenatal testing

For more information, contact us at info@genomicdiagnostics.com.au

1800 822 999 genomicdiagnostics.com.au PO Box 250, Heidelberg West, VIC 3081

Healius Pathology companies

abbott pathology | dorevitch pathology | laverty pathology | qml pathology | tml pathology | western diagnostic pathology

harmony®

AUSTRALIAN Clinicallabs

antenatal.clinicallabs.com.au

Harmony NIPT Request Form

NIPT-02

Patient Information

Patient Name (Surname, Given)

Date of Birth / / Please format DD / MM / YY

Address

City

State Post Code

Phone

Patient Signature for Informed Consent

My signature on this form indicates that I have read, or had read to me, the informed consent and the limitations of this test on the back of this form. I understand the informed consent and give permission to Australian Clinical Labs to perform the laboratory Non-Invasive Prenatal Screening tests selected. In the rare circumstance that Australian Clinical Labs is unable to perform the test, I give permission to Ariosa Diagnostics to perform the Harmony Prenatal Test. I have had the opportunity to ask questions and discuss the capabilities, limitations, and possible risks of the test(s) with my healthcare provider or someone my healthcare provider has designated. I have been informed that 1-2% of tests do not yield a result due to biological factors, and that a second collection may be required. I know that if I wish, I may obtain professional genetic counselling before signing this consent. I will follow the refund or repeat testing policy of Clinical Labs and understand that test results can be delayed in unusual circumstances.

Patient Signature ✗

Date / / Please format DD / MM / YY

Test Menu Options MANDATORY

- ☐ Harmony Prenatal Test (T21, T18, T13) *(please pay online)*
- ☐ Harmony Prenatal Test (T21, T18, T13) + **22q11.2**[1,*] *(please pay online)*

Optional Add On Tests - Please mark any additional test options requested:

- ☐ Fetal Sex **
- ☐ Monosomy X [1,2]**
- ☐ Sex Chromosome Aneuploidy Panel [1,2]**

[1] Singletons only [2] Fetal sex not reported *Additional charge applies **No extra charge

Payment Information MANDATORY

Harmony must be paid prior to your collection.
Please scan the QR Code or visit the website link below to pay for your test online.

pay.clinicallabs.com.au/harmony

Referring Clinician

Name

Address

City

State Post Code

Phone

Fax

Clinician Signature

I attest that my patient has been fully informed about details, capabilities, and limitations of the test(s). The patient has given full consent for this test.

Clinician Signature ✗

Date / / Please format DD / MM / YY

Copy Doctor

Name

Address

Phone Fax

Clinical Information MANDATORY

Gestational Age (10 weeks or above):

- ☐ LMP DD / MM / YY
- ☐ EDD DD / MM / YY

Number of Fetuses ☐ 1 ☐ 2

Conception information:

- ☐ Natural
- ☐ IVF (Patient Egg) | Age at egg retrieval: _____ Years
- ☐ IVF (Donor Egg) | Age at egg retrieval: _____ Years

Maternal information:

Maternal weight (kg) _____ Maternal height (cm)_____

History of high probability of chromosomal abnormality: YES / NO

Important Collector Instructions

- Confirm pre-payment prior to collection.
- Add the Receipt Number to box on right hand side.
- Invert tube 10 times - do not shake.
- Write the patient's full name and date of birth on tube barcodes.
- Name, barcode, and date of birth must match the TRF.
- Place labels lengthwise on the blood tubes as shown in the example:

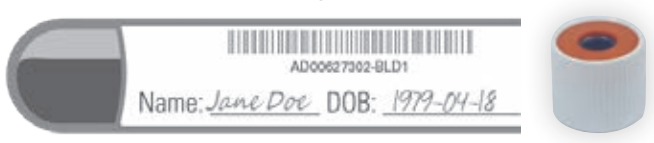

x2

ACC Stamp

Is this a recollect? Yes No

If yes, please collect a gel tube as well as 2 x Harmony tubes if patient is <14 weeks

Write patient receipt number in box below

Receipt Number_______________________________

Time of collection: ___________ Collection Date: DD / MM / YY

Collector Name (please print): ___________________

Collector Signature: ✗ ___________________

Patient Informed Consent

The Harmony Prenatal Test and the available test options are screening tests that analyse cell-free DNA (cfDNA) in maternal blood. The tests aid in the probability determination of some fetal chromosomal or genetic conditions, and fetal sex determination, if selected. In some cases, follow up confirmatory testing based on these tests results could uncover maternal chromosomal or genetic conditions. For a full test description of the Harmony Prenatal Test and available test options, please visit: www.harmonytest.com

Results from the Harmony Prenatal Test should be communicated in a setting designated by your healthcare provider that includes the availability of appropriate genetic counselling.

Who is eligible for the Harmony Prenatal Test?

Patients must be of at least 10 weeks gestational age for any of the Harmony Test offerings. Patients who have received bone marrow or organ transplants or those who have metastatic cancer are not eligible for the Harmony Prenatal Test. Patients who have been identified as having a pregnancy with a demised twin are not eligible for testing. Harmony NIPT has not been validated in demised or vanished twins. False negative or positive results may occur.

Please see below for additional eligibility criteria:

	Harmony (Trisomy 21,18,13) with or without Fetal Sex Option	Harmony with Sex Chromosome Aneuploidy Panel, Monosomy X or 22q11.2
**Singleton Pregnancies including IVF**	✓	✓
**Twin Pregnancies including IVF**	✓	Not eligible
**More than 2 Fetuses**	Not eligible	Not eligible

What are the limitations of the Harmony Prenatal Test?

The Harmony Prenatal Test is not intended nor validated for diagnosis or detection of mosaicism, triploidy, partial trisomy, or translocations. Certain rare biological conditions may also affect the accuracy of the test. Limited numbers of aneuploidy twin and egg donor pregnancies have been evaluated because these conditions are rare. Results for twin pregnancies reflect the probability that the pregnancy involves at least one affected fetus. For twin pregnancies, male results apply to one or both fetuses and female results apply to both fetuses.

Not all trisomy fetuses will be detected. Some trisomy fetuses may have LOW PROBABILITY results. Some non-trisomy fetuses may have HIGH PROBABILITY results. False negative and false positive results are possible. This could be A LOW PROBABILITY result does not guarantee an unaffected pregnancy due to the screening limitations of the test. NIPT screening and confirmatory testing discrepancies are usually due to placental mosaicism. Harmony provides a probability assessment, not a diagnosis, and results should be considered in the context of other clinical criteria. It is recommended that a HIGH PROBABILITY result and/or other clinical indications of a chromosomal abnormality be confirmed through fetal karyotype analysis such as amniocentesis. It is recommended that results be communicated in a setting designated by your healthcare provider that includes appropriate counselling.

What are the limitations of the Harmony Prenatal Test for 22q11.2?

In addition to the limitations discussed above, the 22q11.2 option is not validated for use in pregnancies with more than one fetus or for women with a 22q11.2 duplication or deletion.

A 22q11.2 deletion may not be detected in all affected fetuses. Due to the limitations of the test, a NO EVIDENCE OF A DELETION OBSERVED result does not guarantee that a fetus is unaffected by a chromosomal or genetic condition. Some fetuses with a 22q11.2 deletion may receive a test result of NO EVIDENCE OF A DELETION OBSERVED. Some fetuses without the 22q11.2 deletion may receive a test result of HIGH PROBABILITY OF A DELETION. In cases of HIGH PROBABILITY results and/or other clinical indications of a chromosomal condition, confirmatory testing is necessary for diagnosis.

NIPT 02

What is done with my sample after testing is complete?

No additional clinical testing will be performed on your blood sample other than those authorised by your healthcare provider. Australian Clinical Labs will disclose the test results only to the healthcare provider(s) listed on the front of this form, or to his or her agent, unless otherwise authorised by you or as required by laws, regulations, or judicial order.

Payment / Billing Information

In a small percentage of cases, it will not be possible to generate a Harmony Prenatal Test result. If your first test does not produce a result, you will be asked if you would like to provide a new blood sample for testing. If you choose not to proceed, you are entitled to a full refund. If you decide to supply a new sample for testing, there will be no additional cost to you, and it will be covered by your initial payment. If the second sample does not produce a conclusive result, then a cost recovery may be charged to you. This partial refund will be processed automatically upon a second time fail. ***Any refund issued by Australian Clinical Labs will be processed within 2-3 weeks back to the credit card used to pay at the initial testing stage.*** For pregnant women who still wish to undergo the test for a third time, a full test fee will be incurred, and there will be no entitlement to a refund if the test fails.

Results will be made available to you through your nominated doctor. Genetic counselling will be offered to all women whose test result indicates a pregnancy with high probability of chromosomal abnormality, within 48 business hours. Upon referral from your requesting clinician, an initial telephone consultation with a certified genetic counsellor is available at no additional cost.

Collection Centres

For a current list of centres, please visit our website **antenatal.clinicallabs.com.au**

All of our collection centres across Victoria, New South Wales, Queensland, South Australia, Western Australia, the Northern Territory and the Australian Capital Territory can collect a blood sample for the Harmony prenatal test. For our collection centres in South Australia and the Northern Territory, we recommend calling ahead.

For frequently asked questions about Harmony NIPT testing, please visit our website antenatal.clinicallabs.com.au/patient/harmony/faq or scan the QR code.

harmony®

Roche

AUSTRALIAN Clinicalabs

Non-invasive prenatal testing (NIPT) based on cell-free analysis is not diagnostic. Results should be confirmed by diagnostic testing. Before making any treatment decisions, all women should discuss their results with their healthcare provider, who can recommend confirmatory diagnostic testing where appropriate. The Harmony Prenatal Test is developed by Ariosa Diagnostics and is performed in Australia.

© 2023 Roche Diagnostics.

HARMONY PRENATAL TEST and HARMONY are trademarks of Roche. All other trademarks are the property of their respective owners.

antenatal.clinicallabs.com.au

Sonic Genetics

Service available through Sonic Healthcare practices:

Douglass Hanly Moir Pathology
Sullivan Nicolaides Pathology
Melbourne Pathology
Barratt & Smith Pathology
Capital Pathology
Clinipath Pathology
Bunbury Pathology
Clinpath Pathology
Hobart Pathology
Launceston Pathology
North West Pathology
Southern.IML Pathology

INSTRUCTIONS FOR THE PATIENT

To finalise the booking and payment for your NIPT, please visit **sonicgenetics.com.au/bookandpay**

All enquiries, please contact 1800 010 447 (Monday–Friday, 8 am–5 pm AEST).

Non-invasive prenatal test (NIPT) | Request form

NIPT-03

FOR THE DOCTOR

This test should be requested by the doctor responsible for medical management of a patient's non-invasive prenatal testing.

Patient details

First name ____________________

Surname ____________________

Date of birth ____/____/____ Sex **Female – Pregnant**

Address ____________________

____

____

Phone (mobile) ____________________

Test(s) requested

NIPT for: Trisomy 21, 18, 13 ☑ Yes

OPTIONS (no charge)

Fetal sex* ☐ Yes

Sex chromosome aneuploidy^ (singleton only) ☐ Yes

*Based on the presence or absence of the Y chromosome. For twin pregnancies this could indicate either two females (if absent) or at least one male.
^If sex chromosome aneuploidy is detected, the fetal sex will be revealed.

OPTIONAL SPECIALISED TESTING (additional charge)

Genome-wide NIPT# ☐ Yes

#The screening of autosomal aneuploidies, including gains and losses >7Mb. This option must be selected by the requesting doctor prior to sample collection. This option includes screening for sex chromosome aneuploidy in singleton pregnancies. See overleaf for information before ordering.

Is this a ☐ **RE-COLLECTION?** Previous Lab ID ____________________

Staff ID/Location	☐ 1 x NIPT tube	Date re-collected	Time re-collected	Re-collect PAY CAT
		/ /	:	**SGUN**

Clinical information REQUIRED

This section must be completed for testing to proceed.

Please note: The requested clinical information is essential for test accuracy. If any of the clinical information you provide below needs updating, please notify the laboratory immediately.

NUMBER OF FETUSES

(assumed singleton, unless otherwise indicated)

☐ Twin pregnancy

GESTATIONAL INFORMATION

☐ LMP ____/____/____ (date) *or* ☐ EDC ____/____/____ (date)

The presence of any of the following invalidates the NIPT result; an alternative test should be considered.

- Taken at less than 10 weeks' gestation
- There are three or more fetuses
- There is known presence of a demised fetus
- There is known presence of maternal aneuploidy, maternal transplant or maternal malignancy

NIPT is not a test of fetal viability.

Requesting doctor

Name ____________________

Address ____________________

____

____

Phone ____________________ Provider No. ____________________

I confirm that this patient has been counselled about the purpose, scope and limitations of the test and has given consent.

DOCTOR SIGNATURE

X Date

Copy reports to

Name ____________________

Address ____________________

____

____

FOR THE PATIENT – Patient and Financial Consent

I consent to the non-invasive prenatal test (NIPT) being performed and confirm that I have been advised about the purpose, scope and limitations of the test. I understand that I can request further information or genetic counselling before or after the test. I understand that NIPT is primarily a screen for an extra copy of chromosomes 21, 18 and 13, and can potentially examine other chromosomes as requested by my doctor on this form.

I understand that the result of this test should be interpreted by my doctor in conjunction with other clinical information and tests, and that it should not be the sole basis for making a decision about my pregnancy. I understand that a second blood collection may be required, that a small percentage of tests do not yield a result due to biological factors, and that I can seek a refund if there is no result for chromosomes 21, 18 and 13. A refund is not available if there is no result for sex chromosome abnormalities/fetal sex/other chromosomes.

☐ I do not agree to the laboratory contacting my treating doctors to obtain information and results regarding this pregnancy for quality assurance purposes.

PATIENT SIGNATURE

X Date

Full payment is required prior to sample collection. Medicare benefits do not apply.
Following payment, you will receive an email and SMS confirmation of your booking.
Please make sure to bring this request form and booking confirmation with you on the day.
To locate a collection centre for your NIPT, please visit sonicgenetics.com.au/locations

FOR THE COLLECTOR

I certify that I established the identity of the patient named on this request, collected and immediately labelled the accompanying specimen(s) with the patient's name, DOB and date/time of collection.

Collector initials	☐ 1 x NIPT tube	Patient initials
Location code	Date collected / /	PAY CAT **SGU**
Collection type	Time collected :	

Sonic Genetics is a brand/trademark of Sonic Pathology Australia Pty Ltd ABN 80 000 853 896, a wholly owned subsidiary of Sonic Healthcare Limited ABN 24 004 196 909 APA 906

SONIC HEALTHCARE

SHG-REQ-0005-00.11
ISSUE MAY 2023

Sonic Genetics

Non-invasive prenatal test (NIPT) | Request form

Information for patients

Purpose

The primary purpose of NIPT is to screen for common chromosome conditions that can affect the health of a baby, that is, Down syndrome (trisomy 21), Edwards syndrome (trisomy 18) and Patau syndrome (trisomy 13).

If requested by your doctor, NIPT can also screen for common conditions of the sex chromosomes, that is, Turner syndrome (45,X), Klinefelter syndrome (47,XXY), XXX and XYY in singleton pregnancies. NIPT is not primarily intended as a test of fetal sex.

NIPT can also screen for the gain or loss of other chromosomes or large chromosome regions, that is, genome-wide NIPT. The clinical implications and performance metrics for this option are different to those for the common autosomal trisomies and these should be considered and discussed before having this test. Please refer to sonicgenetics.com.au for further details.

Fetal fraction

The mother's bloodstream contains DNA fragments from both the mother and the developing fetus/placenta. The proportion of DNA fragments from the fetus/placenta is known as the fetal fraction. Numerous factors, including a gestation of less than 10 weeks and high maternal weight, can lower the fetal fraction. If the fetal fraction is too low the NIPT cannot provide a reliable result. Sonic Genetics will not provide a result unless the required quality thresholds are met. In some situations, we may offer to repeat the test (once, and for free) if there is a reasonable chance of providing a result for chromosomes 21, 18 and 13 on the analysis of a new blood sample.

Limitations

There are rare occasions when an NIPT result does not accurately reflect the chromosomal status of the fetus. All NIPTs rely on fragments of DNA found in the mother's bloodstream; these fragments come from the placenta. The placenta and the fetus develop from the same single cell (the fertilised egg) and usually have the same chromosomes. Sometimes, the DNA status of the placenta is not the same as that of the fetus and, as a result, the NIPT result may not reflect the DNA status in the fetus. For these reasons, among others, NIPT is considered a screening test. It is not designed to give a definitive result and all findings should be confirmed with the appropriate diagnostic test. An NIPT result should be carefully reviewed by your doctor, together with other information about your pregnancy, before basing any decision on that result. An ultrasound scan prior to the NIPT is recommended to confirm viability, number of fetuses and gestation. Please also note that NIPT is a test for the conditions noted above; it is not a test for every possible fetal condition or fetal viability. These considerations apply to any form of NIPT.

Fetal sex and sex chromosomes

For biological reasons, it is more difficult to count the numbers of sex chromosomes (X and Y) than other chromosomes. We do not routinely report fetal sex or analysis of sex chromosomes, but will do so on request. Please note that the request should be made by your doctor on the initial request form. Analysis for differences in the number of sex chromosomes is only available for singleton pregnancies.

In a small percentage of samples, a result can be provided regarding the probability of trisomy 21, 18 or 13 but not for fetal sex or sex chromosome conditions (or both). Various factors may make it difficult to provide accurate sex chromosome analysis, including poor quality of DNA in the sample, uncommon normal variations in the sex chromosomes, and a mixture of normal and abnormal cells in the placenta or mother. In these situations, we will report that a result is not possible for fetal sex or screening for a sex chromosome abnormality; alternatively, the accuracy of evaluation for sex chromosome conditions may be compromised. We do not recommend repeat testing, as the biological factors responsible for the lack of a result are unlikely to have changed. We do not offer a refund, as the primary purpose of NIPT (screening for trisomy 21, 18 and 13) has been achieved.

Genetic counselling

Various forms of prenatal screening tests have been available in Australia for more than 30 years. Your doctor will be able to provide you with information and advice regarding this test, and can help you decide if this is the right test for you. Your doctor may also recommend that you seek genetic counselling before or after the test; we can provide contact details for genetic counselling services nationally.

If you have a result indicating a high likelihood of a chromosome condition, a genetic counselling consultation by telephone is available through Sonic Genetics at no charge. This must be requested by your doctor, and is only available to patients who have paid Sonic Genetics directly for their test. Please refer to sonicgenetics.com.au/patient/nipt/gc for further details.

If you have any questions regarding your test result, we recommend that you discuss these with your doctor or genetic counsellor. Your doctor will know your situation and have the results of other tests and assessments. Your doctor can also contact our senior scientists and genetic pathologists to discuss technical aspects of your result.

Results

Your results will be delivered to your doctor typically within 3–8 business days of the laboratory receiving your sample.

NIPT-03

PRENATAL SCREENING REQUEST FORM - TWIN / SINGLETON

Monash IVF Pty Ltd - A.P.A. 1177

MEDICARE NUMBER:

nest

repromed

RCPA The Royal College of Pathologists of Australasia

NATA WORLD RECOGNISED ACCREDITATION

TO MAKE AN APPOINTMENT REFER TO INFORMATION OVERLEAF

180 Fullarton Road Dulwich SA 5065 TEL **1800 874 971** FAX **(08) 8333 8188** A.P.L. 39625 REQUEST FORM

RCPA/NATA ACCREDITATION NO. 2774
APP - Dr. J. Woolcock; Dr. R. Henshaw

PATIENT DETAILS

PATIENT LAST NAME | GIVEN NAME (INCLUDING MIDDLE INITIAL) | DATE OF BIRTH | CLIENT REF. NO.

PATIENT ADDRESS | TELEPHONE | REQUEST NO.

CLINICAL NOTES | WEIGHT (KG) | HEIGHT (CM)

SELF DETERMINED ☐

TEST REQUESTED - NIPT

VIABILITY ULTRASOUND ☐

SINGLETON

NEST Chromosomes 21, 18, 13 ☐

NEST Chromosomes 21, 18, 13 **plus Sex Chromosome Aneuploidies (SCA)** ☐

FETAL GENDER printed on report **(Note: the SCA option must be selected)** ☐

TWINS

NEST Chromosomes 21, 18, 13 ☐

PRESENCE OF Y CHROMOSOME printed on report ☐

Interpretation
No Y chromosome = 2 Female twins
Yes Y chromosome = Either 1 or 2 male twins

ESSENTIAL INFORMATION

Gestational Age (requesting doctor to complete)

☐ LMP ____________ **OR** ☐ EDD ____________ **OR**

☐ BY SCAN Scan Date ____________ ____ wks ____ days

Other essential information

IVF conceived ☐ Ovulation medication ☐

Donor egg ☐ Age of Donor ____ yrs

Have you had a previous Trisomy pregnancy? ☐ Yes ☐ No

If yes, which Trisomy? ____________

COMMENTS

TEST REQUESTED - FIRST TRIMESTER BIOMARKERS

SINGLETON ☐ | TWINS ☐

FIRST TRIMESTER BIOMARKERS (freeBhCG, PAPP-A, PLGF) ☐

Note: Essential information must be completed for freeBhcg and PAPP-A results in MoMs

ESSENTIAL INFORMATION

Gestational Age

CRL ____________ mm Scan date ____________

Ethnicity (patient may complete)

- ☐ Afro-Caribbean (African, Caribbean, African-American)
- ☐ Asian (Indian, Pakistani, Bangladeshi, Sri Lankan, Afghani)
- ☐ Caucasian (European, Middle Eastern, North African, Hispanic)
- ☐ Oriental (Chinese, Korean, Japanese, Malaysian, Indonesian)
- ☐ Other (mixed race)

Other essential information (patient may complete)	Yes	No
Have you been a smoker in this pregnancy?	☐	☐
Do you have diabetes?	☐	☐
Are you taking aspirin in this pregnancy?	☐	☐
Have you had a previous pregnancy with pre-eclampsia?	☐	☐
Did your mother have pre-eclampsia in her pregnancy with you?	☐	☐
Do you have High Blood Pressure?	☐	☐
Do you have Systemic-Lupus?	☐	☐
Do you have Antiphospholipid syndrome?	☐	☐

Hospital status of patient at specimen collection or date of service	YES	NO
Private patient in a private hospital or approved day hospital facility	☐	☐
Private patient in a recognised hospital	☐	☐
Public patient in a recognised hospital	☐	☐
Outpatient of a recognised hospital	☐	☐

REQUESTING DOCTOR (PROVIDER NUMBER, SURNAME & INITIALS, ADDRESS)

COPY REPORTS TO

DOCTOR'S SIGNATURE AND REQUEST DATE

X ____________ X ____________

Your treating practitioner has recommended that you use Repromed Laboratories i.e. NEST. You are free to choose your own pathology provider. However, if your doctor has specified a particular pathologist on clinical grounds, a Medicare rebate will only be payable if that pathologist performs the service. You should discuss this with your doctor.

Medicare Assignment
(Section 20A of the Health Insurance Act 1973). I offer to assign my right to benefits to the approved pathology practitioner who will render the requested pathology service(s) and any eligible pathologist determinable service(s) established as necessary by the practitioner.

Informed Consent My signature on this form indicates that I have read or have had read to me the information about the nest test and I consent to having the test performed on my blood. I understand that this test is a screening test for selected abnormalities of chromosomes 21, 18 and 13. In addition, I understand that I can also request to have the sex chromosomes tested which can screen for less serious selected abnormalities of the sex chromosomes and I can also elect to have fetal gender reported. I have had the opportunity to ask questions and discuss limitations of the test with my health care provider or someone that my healthcare provider has designated. I understand that should my test come back with a 'high probability' finding that this result should be confirmed by further testing (chorionic villus sampling or amniocentesis). I also understand that sometimes this testing is unable to provide a result due to biological factors and in this instance I will be provided with a refund.

ATTENTION: DOCTORS/NURSES/PHLEBOTOMISTS

DECLARATION: I certify that I collected the accompanying sample from the above patient whose identity was confirmed by enquiry and/or examination of their name band and that I labelled the sample immediately following collection.

COLLECTOR'S NAME ____________ DATE ____________

COLLECTOR'S SIGNATURE ____________ TIME ____________

PATIENT CONFIRMATION OF CORRECT PERSONAL DETAILS LISTED ON FORM AND INFORMED CONSENT FOR **NEST** TESTING (read informed consent section above).

X ____________ X ____________

PATIENT'S SIGNATURE | DATE

PRACTITIONER'S USE ONLY (REASON PATIENT CANNOT SIGN)

NEST SA_V02

Patients should be aware that they will be invoiced. For prenatal cytogenetic testing patients should note that results will be issued to themselves or their partner unless we are otherwise instructed. FTS is accredited for compliance with NPAAC Standards and ISO 15189.

Privacy Note: The information provided will be used to assess any Medicare benefit payable for the services rendered and to facilitate the proper administration of government health programs, and may be used to update enrolment records. Its collection is authorised by provisions of the Health Insurance Act 1973. The information may be disclosed to the Department of Health and Ageing or to a person in the medical practice associated with this claim, or as authorised/required by the law.

INFORMATION STATEMENT

Monash IVF Pty Ltd - A.P.A. 1177

repromed

nest

repromed RCPA NATA WORLD RECOGNISED ACCREDITATION

RCPA/NATA ACCREDITATION NO. 2774
APP - Dr. J. Woolcock; Dr. R. Henshaw

180 Fullarton Road Dulwich SA 5065 TEL **1800 874 971** FAX **(08) 8333 8188** A.P.L. 39625 **REQUEST FORM**

NEST BLOOD COLLECTION

General information:

- Please make an appointment by phoning 8333 8172
- Appointments may be made at:
  Repromed Dulwich: 180 Fullarton Rd, Dulwich SA 5065
  Repromed Mawson Lakes: 8B Light Common, Mawson Lakes, SA 5095
- Upon arrival please report to the reception desk and notify them that you are here for a NEST blood test
- You will be asked to take a seat in the waiting room and a staff member will collect your blood as soon as possible.
- No fasting is required for your NEST blood test

NEST BLOOD COLLECTION AND ULTRASOUND SERVICE

General information:

- **By appointment only**, please make an appointment by phoning 8333 8172
- Appointments may be made at:
  Repromed Dulwich: 180 Fullarton Road, Dulwich SA 5065
- Upon arrival please report to the reception desk and notify them that you are here for a nest blood test and ultrasound
- You will be asked to take a seat in the waiting room and a staff member will attend to you as soon as possible.
- No fasting is required for your NEST blood test

NOTE:

For your ultrasound please present with a **FULL BLADDER**, ie: you should not pass urine for 1 hour before the examination, then drink 2 glasses of **NON GASEOUS FLUID 1 hour** before the examination. Your Sonographer may advise you that a transvaginal scan may be required, this will only be performed with your consent.

PATIENT ADVISORY STATEMENT:

You have been referred by your doctor to Repromed to ensure you receive the highest quality personalised imaging. You may however consider alternative options with your doctor.

NIPT 04

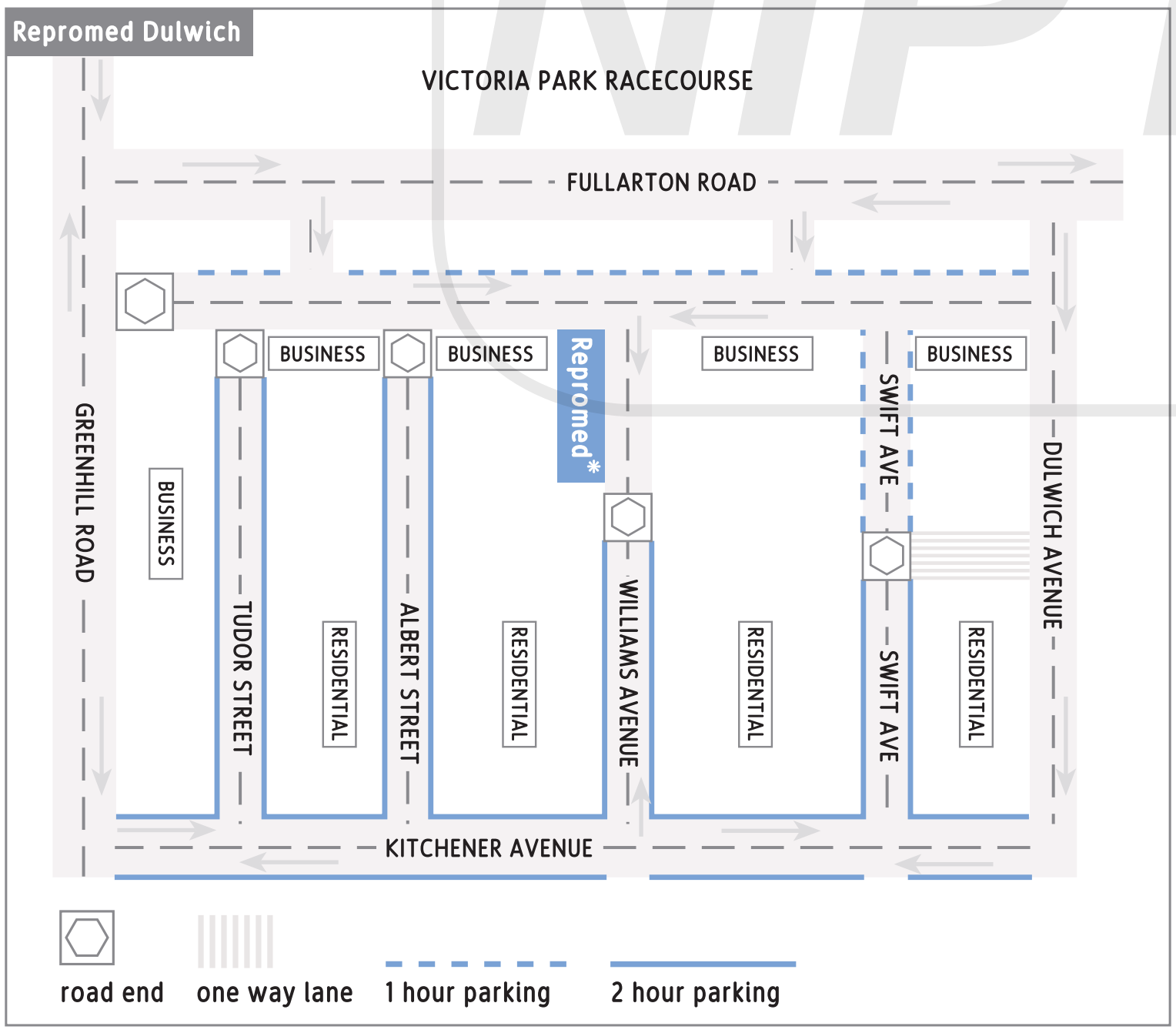

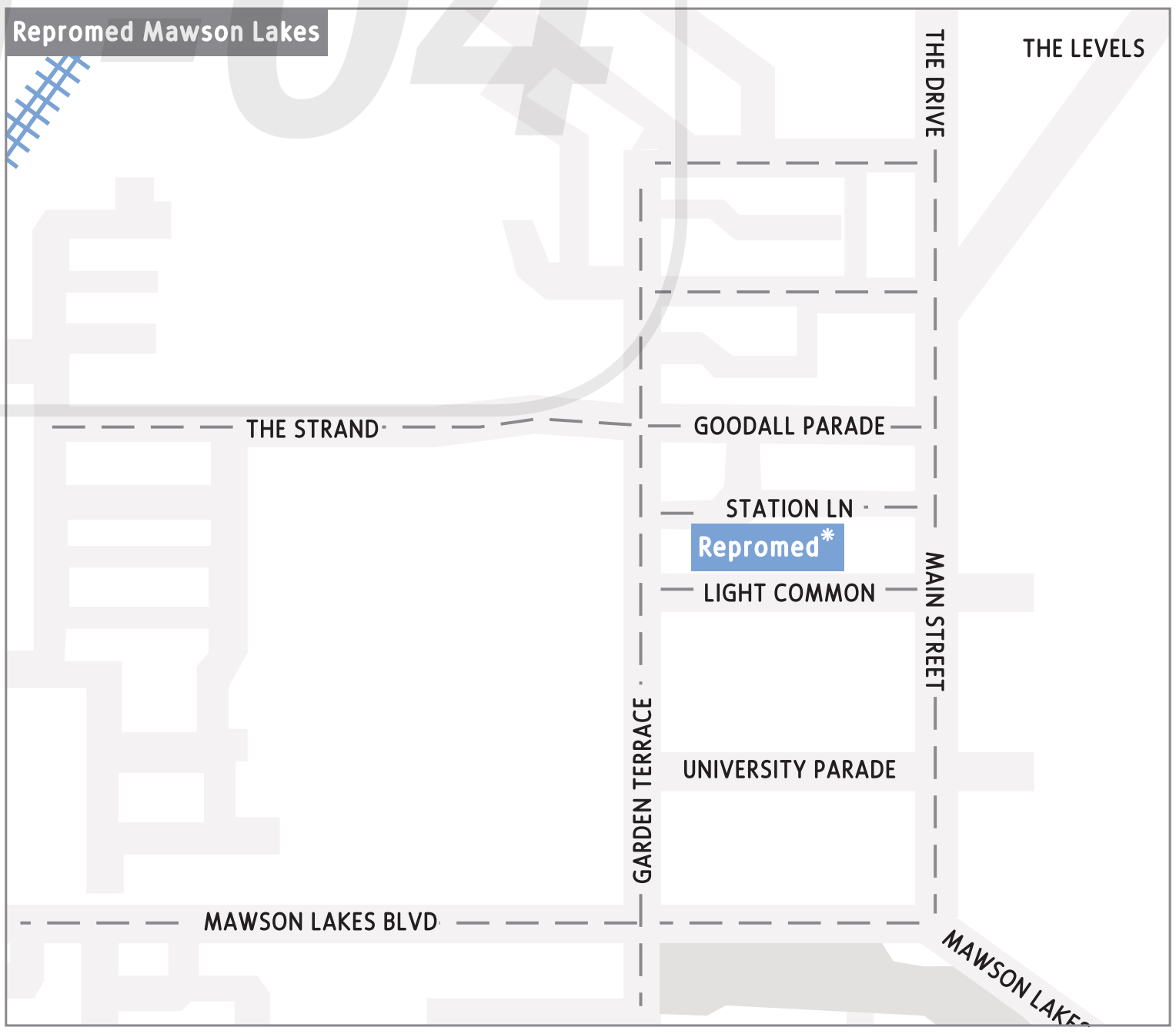

APPOINTMENT DETAILS

repromed 180 Fullarton Road Dulwich SA 5065
TEL **1800 874 971** FAX **(08) 8333 8188**

nest

MEDICARE NUMBER:

DATE OF APPOINTMENT

TIME

LOCATION (TICK ONE)

- ☐ Dulwich
- ☐ Mawson Lakes

TEST REQUIRED (TICK ONE)

- ☐ NEST blood test only
- ☐ NEST blood test and ultrasound

PRENATAL SCREENING REQUEST FORM - SINGLETON

Monash IVF Pty Ltd - A.P.A. 1177

nest+ | repromed | RCPA | NATA WORLD RECOGNISED ACCREDITATION

MEDICARE NUMBER:

TO MAKE AN APPOINTMENT REFER TO INFORMATION OVERLEAF

180 Fullarton Road Dulwich SA 5065 TEL **1800 874 971** FAX **(08) 8333 8188** A.P.L. **39625** REQUEST FORM

RCPA/NATA ACCREDITATION NO. 2774*
APP - Dr. J. Woolcock; Dr. R. Henshaw

PATIENT DETAILS

PATIENT LAST NAME | GIVEN NAME (INCLUDING MIDDLE INITIAL) | DATE OF BIRTH | CLIENT REF. NO.

PATIENT ADDRESS | TELEPHONE | REQUEST NO.

CLINICAL NOTES | WEIGHT (KG) | HEIGHT (CM)

SELF DETERMINED ☐

TEST REQUESTED - NIPT

VIABILITY ULTRASOUND ☐

SINGLETON

*NEST All chromosomes ☐

*NEST All chromosomes **plus SCA [Sex Chromosome Aneuploidies (SCA)]** ☐

FETAL GENDER printed on report
(Note: the SCA option must be selected) ☐

ESSENTIAL INFORMATION

Gestational Age (requesting doctor to complete)

☐ LMP ______ **OR** ☐ EDD ______ **OR**

☐ BY SCAN Scan Date ______ wks ______ days

Other essential information

IVF conceived ☐ Ovulation medication ☐

Donor egg ☐ Age of Donor ______ yrs

Have you had a previous Trisomy pregnancy? ☐ Yes ☐ No

If yes, which Trisomy? ______

COMMENTS

TEST REQUESTED - FIRST TRIMESTER BIOMARKERS

FIRST TRIMESTER BIOMARKERS (freeBhCG, PAPP-A, PLGF) ☐

Note: Essential information must be completed for freeBhcg and PAPP-A results in MoMs

ESSENTIAL INFORMATION

Gestational Age

CRL ______ mm Scan date ______

Ethnicity (patient may complete)

☐ Afro-Caribbean (African, Caribbean, African-American)
☐ Asian (Indian, Pakistani, Bangladeshi, Sri Lankan, Afghani)
☐ Caucasian (European, Middle Eastern, North African, Hispanic)
☐ Oriental (Chinese, Korean, Japanese, Malaysian, Indonesian)
☐ Other (mixed race)

Other essential information (patient may complete)	Yes	No
Have you been a smoker in this pregnancy?	☐	☐
Do you have diabetes?	☐	☐
Are you taking aspirin in this pregnancy?	☐	☐
Have you had a previous pregnancy with pre-eclampsia?	☐	☐
Did your mother have pre-eclampsia in her pregnancy with you?	☐	☐
Do you have High Blood Pressure?	☐	☐
Do you have Systemic-Lupus?	☐	☐
Do you have Antiphospholipid syndrome?	☐	☐

Hospital status of patient at specimen collection or date of service	YES	NO
Private patient in a private hospital or approved day hospital facility	☐	☐
Private patient in a recognised hospital	☐	☐
Public patient in a recognised hospital	☐	☐
Outpatient of a recognised hospital	☐	☐

REQUESTING DOCTOR (PROVIDER NUMBER, SURNAME & INITIALS, ADDRESS)

COPY REPORTS TO

DOCTOR'S SIGNATURE AND REQUEST DATE

X ______ X ______

Your treating practitioner has recommended that you use Repromed Laboratories i.e. NEST. You are free to choose your own pathology provider. However, if your doctor has specified a particular pathologist on clinical grounds, a Medicare rebate will only be payable if that pathologist performs the service. You should discuss this with your doctor.

Medicare Assignment
(Section 20A of the Health Insurance Act 1973). I offer to assign my right to benefits to the approved pathology practitioner who will render the requested pathology service(s) and any eligible pathologist determinable service(s) established as necessary by the practitioner.

Informed Consent My signature on this form indicates that I have read or have had read to me the information about the nest test and I consent to having the test performed on my blood. I understand that this test is a screening test for selected abnormalities of chromosomes 1-22. In addition, I understand that I can also request to have the sex chromosomes tested which can screen for less serious selected abnormalities of the sex chromosomes and I can also elect to have fetal gender reported. I have had the opportunity to ask questions and discuss limitations of the test with my health care provider or someone that my healthcare provider has designated. I understand that should my test come back with a 'high probability' finding that this result should be confirmed by further testing (chorionic villus sampling or amniocentesis). I also understand that sometimes this testing is unable to provide a result due to biological factors and in this instance I will be provided with a refund.

ATTENTION: DOCTORS/NURSES/PHLEBOTOMISTS

DECLARATION: I certify that I collected the accompanying sample from the above patient whose identity was confirmed by enquiry and/or examination of their name band and that I labelled the sample immediately following collection.

COLLECTOR'S NAME ______ DATE ______

COLLECTOR'S SIGNATURE ______ TIME ______

PATIENT CONFIRMATION OF CORRECT PERSONAL DETAILS LISTED ON FORM AND INFORMED CONSENT FOR **NEST+** TESTING (read informed consent section above).

X ______ X ______

PATIENT'S SIGNATURE | DATE

PRACTITIONER'S USE ONLY (REASON PATIENT CANNOT SIGN)

NEST+ SA_2021v2

Patients should be aware that they will be invoiced. For prenatal cytogenetic testing patients should note that results will be issued to themselves or their partner unless we are otherwise instructed. FTS is accredited for compliance with NPAAC Standards and ISO 15189.

Privacy Note: The information provided will be used to assess any Medicare benefit payable for the services rendered and to facilitate the proper administration of government health programs, and may be used to update enrolment records. Its collection is authorised by provisions of the Health Insurance Act 1973. The information may be disclosed to the Department of Health and Ageing or to a person in the medical practice associated with this claim, or as authorised/required by the law.

NIPT-05

INFORMATION STATEMENT

Monash IVF Pty Ltd - A.P.A. 1177

repromed

nest+

repromed RCPA NATA

180 Fullarton Road Dulwich SA 5065 TEL **1800 874 971** FAX **(08) 8333 8188** A.P.L. **39625** REQUEST FORM

RCPA/NATA ACCREDITATION NO. 2774
APP - Dr. J. Woolcock; Dr. R. Henshaw

NEST BLOOD COLLECTION

General information:

- Please make an appointment by phoning 8333 8172
- Appointments may be made at:
  Repromed Dulwich: 180 Fullarton Rd, Dulwich SA 5065
  Repromed Mawson Lakes: 8B Light Common, Mawson Lakes, SA 5095
- Upon arrival please report to the reception desk and notify them that you are here for a NEST blood test
- You will be asked to take a seat in the waiting room and a staff member will collect your blood as soon as possible.
- No fasting is required for your NEST blood test

NEST BLOOD COLLECTION AND ULTRASOUND SERVICE

General information:

- **By appointment only**, please make an appointment by phoning 8333 8172
- Appointments may be made at:
  Repromed Dulwich: 180 Fullarton Road, Dulwich SA 5065
- Upon arrival please report to the reception desk and notify them that you are here for a nest blood test and ultrasound
- You will be asked to take a seat in the waiting room and a staff member will attend to you as soon as possible.
- No fasting is required for your NEST blood test

NOTE:

For your ultrasound please present with a **FULL BLADDER**, ie: you should not pass urine for 1 hour before the examination, then drink 2 glasses of **NON GASEOUS FLUID 1 hour** before the examination. Your Sonographer may advise you that a transvaginal scan may be required, this will only be performed with your consent.

PATIENT ADVISORY STATEMENT:

You have been referred by your doctor to Repromed to ensure you receive the highest quality personalised imaging. You may however consider alternative options with your doctor.

NIPT-05

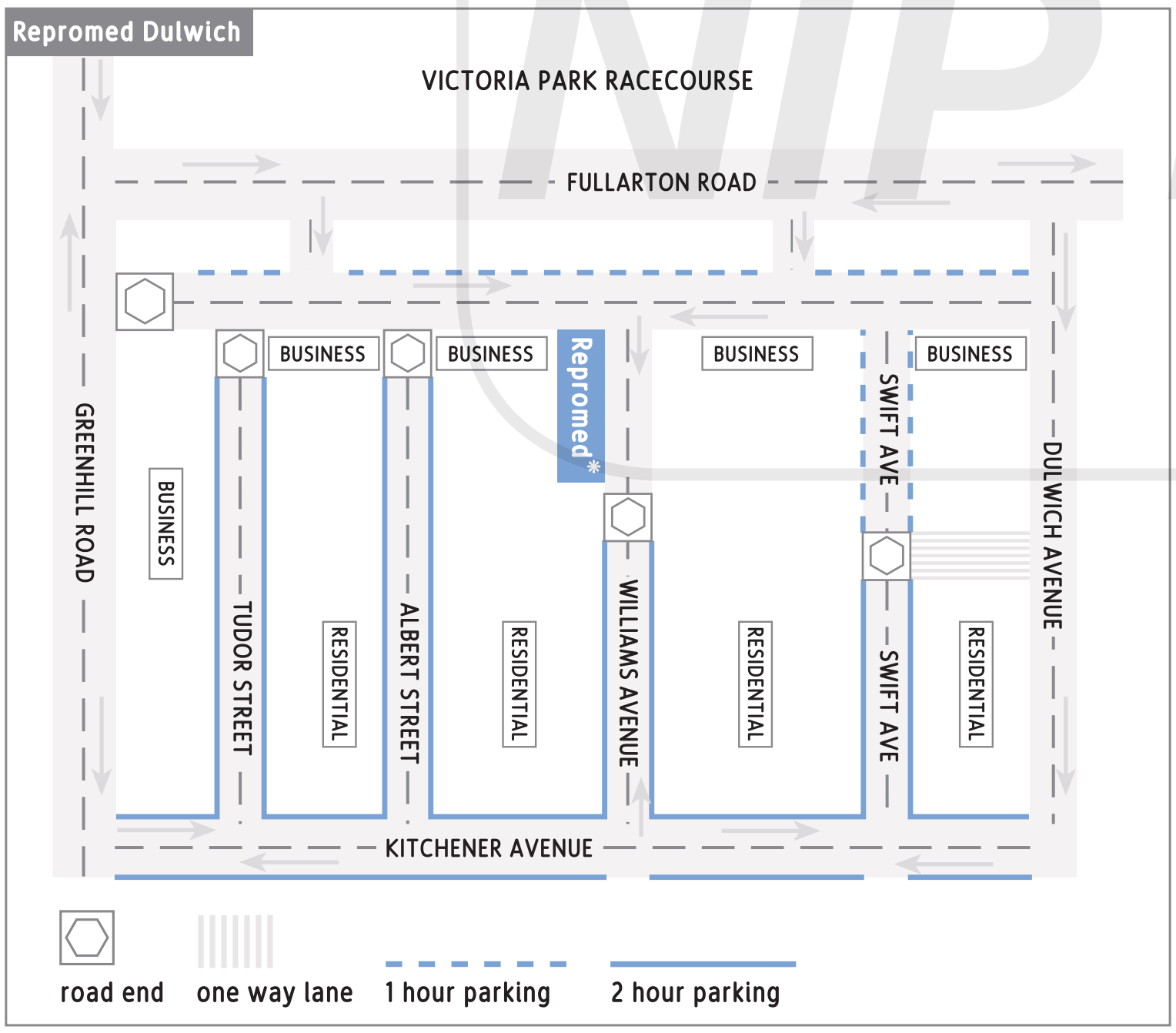

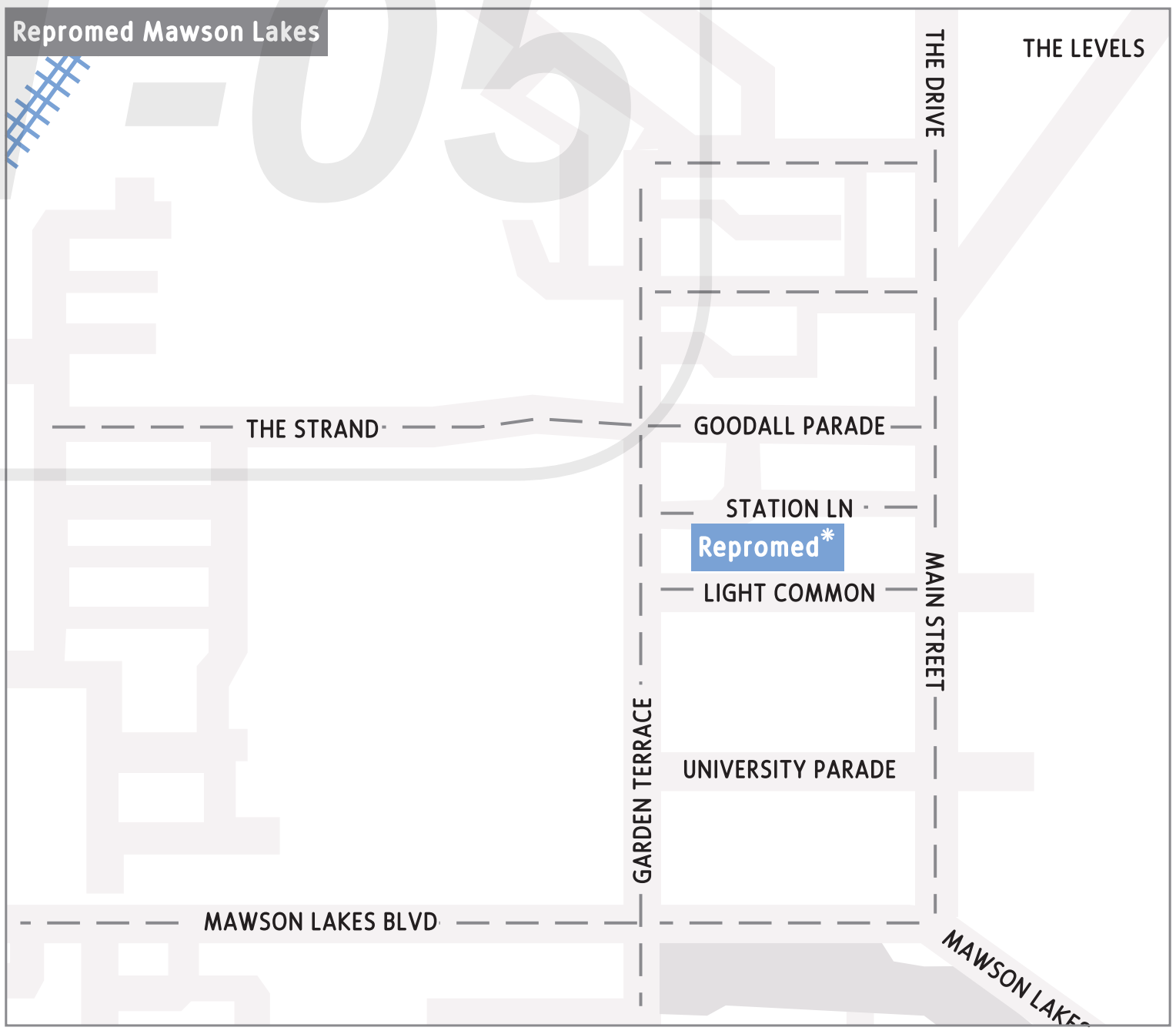

APPOINTMENT DETAILS

repromed 180 Fullarton Road Dulwich SA 5065
TEL **1800 874 971** FAX **(08) 8333 8188**

nest

MEDICARE NUMBER:

DATE OF APPOINTMENT

TIME

LOCATION (TICK ONE)

☐ Dulwich

☐ Mawson Lakes

TEST REQUIRED (TICK ONE)

☐ NEST blood test only

☐ NEST blood test and ultrasound

Genomics for life

NIFTY™ Non-Invasive Fetal TrisomY test

NON-INVASIVE PRENATAL GENETIC SCREENING REQUEST FORM

ilac-MRA NATA Accreditation No. 19325

Accredited for compliance with NPAAC Standards and ISO 15189. NATA Accreditation No: 19325

NIPT-06

PATIENT DETAILS

Patient Surname:

Patient Given Names:

DOB: ____ / ____ / ______

Patient Address:

Contact Number:

Weight (Kg): __________ Height (cm): __________ BMI: ________

Email:

☐ Please notify me via email when the results have been sent to my referring doctor.

Patient Informed Consent Statement

I have read, understood, and agree to the attached Informed Consent Statement, and fully understand the indication, intended purpose, limitations, and potential risks of this test, as explained by my physician. I confirm the personal information I have provided is true and correct. Please note that some terms in the attached Informed Consent require a 'Yes' or 'No' response.

Patient Signature: **Date:**

ORDERING MEDICAL PRACTITIONER DETAILS

Doctor Name:

Provider Number:

Practice Name:

Practice Address:

Contact Number:

Fax or email (for results):

Copy Doctor(s):

Medical Practitioner Statement

By signing this request form, I confirm that I have patient consent for the testing to be performed and confirm that the patient understands the purpose, scope and performance of the screen explained by either myself, available patient literature or the Genomics For Life website.

Doctor Signature: **Date:**

REQUESTED SCREENING

☐ NIFTY™ Plus (DX0609)* - includes T21, T18, T13, selected Sex Chromosome abnormalities & gender identification.

☐ NIFTY™ Plus Twin (DX1447) - includes T21, T18, T13 & gender identification.

☐ NIFTY™ Pro (DX1623)* - includes T21, T18, T13, T9, T16, T22, selected Sex Chromosome abnormalities & 84 Microdeletion/Microduplication Syndromes.

☐ NIFTY™ Single Gene Disease Screening* - includes screening for disease causing variants in 18 genes.

☐ NIFTY™ Screening Bundle* - includes both the NIFTY™ Pro Screening and NIFTY™ Single Gene Disease Screening

Please see our website for a full list of inclusions for each screening option.

**Please note, these tests are not available for twin pregnancies*

FETAL GENDER

☐ Please tick if you WOULD like to know fetal gender

CLINICAL INFORMATION

Gestational Age: ____ weeks ____ days   As At date: ____ / ____ / ____

Estimated Due Date: ____ / ____ / ____

No of Fetus: ☐ Singleton ☐ Twin*

*Please note screening for T21, T18 and T13 only is available for Twin pregnancies.

If Twin: ☐ DCDA ☐ MCDA ☐ MCMA ☐ Unsure

IVF: ☐ No ☐ Yes - Implantation Date: ___ / ___ / _______

Prior Down Syndrome Screening Test:

☐ No ☐ Yes – estimated risk of T21: 1/___, T18: 1/___, T13: 1/____

First Sampling: ☐ Yes ☐ No - Date of first collection ___ / ___ / _______

PATIENT MEDICAL HISTORY

Received allogenic blood transfusion within the past 12 months	☐ No ☐ Yes
Transplant surgery	☐ No ☐ Yes
Received immunotherapy in the past 4 weeks	☐ No ☐ Yes
Stem cell therapy	☐ No ☐ Yes
Heparin therapy	☐ No ☐ Yes
Vanishing twin syndrome	☐ No ☐ Yes
The patient has a single-gene disease	☐ No ☐ Yes - Please specify:
Family history of single gene disease or other genetic disease/s	☐ No ☐ Yes - Please specify:
History of tumours (except maligant tumours during pregnancy)	☐ No ☐ Yes - Please specify:
Taking Medicine During Pregnancy	☐ No ☐ Yes - Please specify:
Abnormal Reproductive History	☐ No ☐ Yes - Please specify:
Abnormal Maternal Karyotype	☐ No ☐ Yes - Please specify:

Please attach any further relevant medical history

Collection Staff Only		Laboratory Use Only	
Date Collect:	Collectors Signature:	Date Rec:	Signature:
Time Collect:		Time Rec:	Comments:

NON-INVASIVE PRENATAL GENETIC SCREENING - INFORMED CONSENT

1. This test is intended to detect fetal Trisomy 21, 18 and 13 for both singleton and twin pregnancies. This test is highly accurate, with a detection rate over 99% and a false positive rate of less than 1% for fetal Trisomy 21, 18, and 13. Due to the limitation of the current technology, a negative result cannot totally exclude the possibility of fetal trisomy. It is also possible to use this test to discover specific sex chromosome aneuploidies and other chromosomal numeric abnormalities, specific locus relevant to 84 kinds of microdeletion/duplication syndromes (please see our website for a full list).
2. It is strongly advised that the NIFTY® test is performed from 10 to 24 gestational weeks of pregnancy. If the test is performed at very early pregnancy stage (<10 gestational weeks), there is a higher risk of a recollection being required due to a low fetal fraction and false-negative results. Therefore, s**amples with gestational week <10 will not be accepted, and a recollection will be required.** Testing may be carried out after 24 gestational weeks only in accordance with local law.
3. NIFTY is NOT a diagnostic test and the result cannot be used as the sole evidence for a diagnostic conclusion. A high-risk result should be followed by confirmatory diagnostic testing, and test report should be interpreted by physician.
4. Although highly accurate, this test cannot be considered a diagnostic test. Abnormalities caused by chromosomal polyploid (triploid, tetraploid, etc), chromosomal balanced translocation, inversion, ring, UPD, monogenic/polygenic disease, etc, cannot be detected by this test; this test cannot exclude the fetal mosaic chromosomal diseases.
5. The high sensitivity and specificity of NIFTY test are based on studies in singleton pregnancies. Based on reported studies so far, and on theoretical grounds, the performance of NIFTY in twin pregnancies is similar to that in singleton pregnancies. **Screening for T21, T18 and T13 only is available for twin pregnancies.**
6. Patients who have received a blood transfusion within one year prior to testing date, transplant surgery or stem cell therapy as well as heparin therapy, are not eligible for the NIFTY® test. We are unable to accept samples in cases of 'vanishing twin syndrome' where developmental arrest has been identified as occurring after week 8 of pregnancy, or within 8 weeks prior to NIFTY® testing date
7. Potential sources of false positive or false negative results and patients who are not suitable for the test include but are not limited to: maternal, fetal and/or placental mosaicism (mixtures of chromosomally normal and abnormal cells in the pregnancy), chromosomal abnormality in either parent, transplant surgery, stem cell therapy, blood transfusion within one year, cellular immunotherapy where exogenous DNA is introduced within 4 weeks, malignant tumor during pregnancy, >2 fetus and low fetal fraction, patient's BMI>40 or if the patient has taken specific medications, such as Heparin, during pregnancy. Gender identification can be false if the detected value is within the gray zone.
8. Reports will be available within 10 - 12 working days from the time the laboratory receives the sample. I understand that my sample may be sent abroad for analysis at a BGI owned and operated laboratory. In a small number of cases, samples may fail Quality Control checkpoints. Resampling in these cases are needed; there is no additional cost for resampling and the turnaround time will be prolonged.
9. Unused test material is important for researching biological mechanisms and quality assurance on genetic tests in the lab. I agree to the anonymous storage and use of remaining test material. Patients can withdraw the test at any time through a written statement, and the sample/s submitted as well as data will then be destructed (data that has been anonymous cannot be withdrawn or deleted).

☐ **Yes** ☐ **No**

10. The laboratory may contact the patient and/or their referring healthcare provider for further information regarding the pregnancy and all previous tests such as ultrasound/other screening/diagnostic tests performed during the pregnancy, in particular if my baby is subsequently found with a chromosomal or genetic disease. Clinical information may be used by the laboratory for the purpose of auditing, quality assurance and research. All data will remain anonymous and unidentifiable during data analysis and any personal information will be removed from any reports or publications.
11. There is a possibility for the recognition of incidental findings that are not necessarily related to the reason for ordering the test. These findings can provide information that was not anticipated and that are unrelated to the individual's reported clinical features but can be of medical value for patient care. I consent for information regarding genetic results that are not necessarily related to the specific reason for which the healthcare provider has ordered the test, to be reported to the referring healthcare provider.

☐ **Yes** ☐ **No**

12. Results are sent to the undersigned healthcare provider. Due to the complexity and implications of the test results, results will only be reported to the referring healthcare provider. Patients should contact their healthcare provider to obtain the results of the test.

Supplemental Terms for NIFTY Single-Gene Disease Screening

1. The NIFTY® Single-Gene screening is a non-invasive prenatal test used to screen the fetus for disease-causing variants in 18 genes (please see our website for a full list). Our test only includes certain genes and corresponding loci, mutation sites and genes not listed are not included in our testing scope. This detection may not cover all possible pathogenic or likely pathogenic variants in a given gene and can't exclude the possibility of other undetected diseases. This test is not intended to provide a final diagnosis and should, in case of a positive result, not be relied on as sole evidence for a diagnostic conclusion. I understand that my sample may be sent abroad for analysis at a BGI owned and operated laboratory.
2. Numerical abnormalities (trisomy, haploid, triploid, tetraploid, and other polyploids), chromosomal partial monomer (deletion), chromosomal partial trisomy (repeated), translocation, inversion, ring, mosaic, uniparental diploid (UPD), genomic imprinting, polygenic disease, recessive monogenic disease, other dominant single-gene disease caused by mutations in the targeted gene cannot be detected by this test. This test cannot identify fetal mosaic dominant monogenic disease, highly repetitive regions and the regions interfered with pseudogenes.
3. Test results can also be used for research and to improve the diagnosis and treatment of genetic diseases. I consent to the storage and use of my anonymous test results in a statistical database for scientific purposes and to facilitate and improve the diagnosis of genetic changes and diseases in other patients. I understand that I will remain anonymous and unidentifiable during data analysis and that any personal information will be rendered non-personal beforehand if used for any reports or publications.

☐ **Yes** ☐ **No**

For full list of remarks and disclaimers, validation publications and test sensitivities please visit genomicsforlife.com.au

NIPT-06

Place ULTRA barcode here

Place NATERA collection kit barcode here

Non Invasive Prenatal Test (NIPT) Pathology Request

1 PATIENT INFORMATION

Patient Name (Last, First): ____________________

DOB (DD/Month/YYYY): ____________________

Address: ____________________

City: ____________________ State: __________

Post Code: ________ **AUSTRALIA** Phone: +61 ____________

Email: ____________________

Weight: (kg): __________ **Height:** (cm): __________

What stage of pregnancy is patient at?

☐ 1st Trimester ☐ 2nd Trimester ☐ 3rd Trimester

Due Date (DD/Month/YYYY) ____________________

Patient must be at least 9 weeks gestation. 22q is not available for dizygotic twins or egg donors. Extended panel is not available for twins or egg donors.

For twin or surrogate pregnancies, check all that apply. We do NOT accept vanished twin, higher order multiple gestation pregnancies with more than 2 fetuses, or twins conceived using a surrogate or egg donor.

☐ **IVF conceived pregnancy:** Age of mother at egg retrieval __________

☐ **Ongoing twin pregancy:** ☐ Monochorionic ☐ Dichorionic ☐ Don't know

☐ Surrogate or egg donor pregnancy

Patient Signature & Date: ____________________ (DD ___ / Month ___ / YYYY ___)

2 ORDERING CLINICIAN

Ordering Clinician: ____________________

Provider Number: ____________________

Address: ____________________

City: ____________________ State: __________

Post Code: ________ **AUSTRALIA** ____________________

Phone: +61 ____________ Fax: +61 ____________

Clinician Consent I confirm that all information on this form is accurate and complete to the best of my knowledge and that I have explained the benefits and limitations of the Natera Panorama NIPT to the patient to the best of my ability. I hereby order the Natera Panorama NIPT for this patient.

Clinician Signature: ____________________

Date (DD/Month/YYYY): ______ / ______ / ______

STATE DETAILS (SATELLITE CLINIC)

Indicate which state the sample has been collected to identify which satellite clinic the results/MI will be issued to:

☐ New South Wales / Australian Capital Territory – 7102371
Fax: +61 2 9770 2111

☐ Queensland – 7102375
Fax: +61 7 3015 3051

☐ Victoria / Tasmania – 7102380
Fax: +61 3 9473 4459

Attention accessioning:

If this section is not filled out to identify Satellite Clinic, default will be NSW/ACT

3 SCREENING OPTIONS

Select ONE screening panel option below:

☐ **PANORAMA PRENATAL PANEL +22q11.2 DELETION**
Chromosomes 13, 18, 21, X&Y; Triploidy; 22q11.2 deletion

☐ **PANORAMA EXTENDED PANEL**
Chromosomes 13, 18, 21, X&Y; Triploidy; 22q11.2 deletion PLUS four microdeletions (1p36 deletion, Angelman, Prader-Willi, Cri-du chat)

☐ Tick to **ADD FETAL SEX** to Report (available with any screening option above)

4 SAMPLE COLLECTION DETAILS

Collector Statement: All sections of the request form have been checked and the identity of the patient(s) was verified and the sample(s) labelled immediately after collection.

Date of Blood Draw (DD/Month/YYYY): ____________________

Time of Blood Draw: ____________________

Number of blood sample tubes collected: __________ (2 x 10ml required)

Collection Location: ____________________

Collector's INITIALS: __________ & Signature: ____________________

Disposition or retention of samples: Laboratory (Reseller) represents and confirms that the patient has given informed consent in compliance with applicable law to Natera's following sample disposition or retention policy. PATIENT UNDERSTANDS AND CONSENTS THAT: (i) her/his sample will be sent to the United States for performance of the test; (ii) Natera may retain the patient's leftover, de-identified samples to use for medical and technology advancement, research & development, product validation and quality assurance, independently or in collaboration with third party partners, either in or outside the United States; and (iii) patient and patient's heirs will not receive any payments, benefits or rights to any resulting products or discoveries.

NATERA LIMS ID LAB 2284

5 BOOK AN APPOINTMENT

For your nearest collection centre visit our website at:
www.virtusdiagnostics.com.au/collection-centres

virtus DIAGNOSTICS

panorama™
NEXT GENERATION NIPT
POWERED BY NATERA®

Non Invasive Prenatal Test (NIPT)
Pathology Request Patient Information

This is a non-invasive blood test which studies the fetal DNA in the mother's blood and can be done from 9 to 39 weeks pregnancy. The fetal DNA tested comes from the placenta; this DNA is identical to the DNA found in the actual cells of the fetus in about 98% of all pregnancies. The condition where the DNA in the placenta is different to that of the fetus is known as confined placental mosaicism.

The purpose of the Panorama Non-Invasive Prenatal Test (NIPT) is to screen the fetus for the chromosome abnormalities, including the specific whole extra or missing chromosomes 13,18, 21, X and Y plus optionally specified microdeletions (small missing sections of specified chromosomes). You have the option of requesting a screen and reporting of the fetal sex as well. This screen will therefore detect common chromosome problems such as Down syndrome that may lead to a child born with degrees of mental impairment.

It does NOT detect:

- Any other cause for mental impairment in a child
- Disorders of any other chromosomes, that may lead to an early miscarriage
- Any other cause for birth defects

NIPT-07

The test takes up to two weeks to perform. At that time you will receive one of the following results:

High Risk: There is an increased likelihood that your fetus has an abnormality of one of the above chromosomes and further investigation is strongly recommended.

Low Risk: There is a reduced likelihood that your fetus has an abnormality of one of the above chromosomes.

No result: This can happen when there is insufficient fetal DNA to give a clear result. The test would then need to be repeated (at no cost to you) and this would add 2 weeks onto your gestation period by the time you receive a new result. A high BMI will increase the likelihood of a low/insufficient fetal DNA content.

A "No result" may also happen if the parents are related or the mother's parents are related. Also when there is a multiple pregnancy or vanishing twin or for any other reason the DNA pattern is not clear. A repeat test in these instances is unlikely to generate a result and you will be offered a refund.

The result will be sent back to your referring doctor who will contact you to let you know.

With confined placental mosaicism affecting 1 – 2% of pregnancies, an incorrect high- or low- risk result can occur. The Panorama screen is not a diagnostic test – it will not confirm any of these chromosome abnormalities. It will only provide the risk for each of these in your current pregnancy. Therefore, **DECISIONS ABOUT YOUR PREGNANCY SHOULD NEVER BE MADE BASED ON THESE SCREENING RESULTS ALONE AS THEY NEITHER CONFIRM NOR RULE OUT THE PRESENCE OF A CHROMOSOME ABNORMALITY IN THE FETUS.**

It is the responsibility of your doctor ordering this test to understand the reliability of the test results, the limitations and the alternatives and to explain them to you. Before you commence with the test and sign this form, please ask your doctor for more information about the test and the results if required.

VCGS leaders in genetic health
percept nipt

View collection sites

Victorian Clinical Genetics Services
The Royal Children's Hospital
Flemington Road, Parkville VIC 3052
P +61 1300 118 247 **F** +61 3 8341 6366
W vcgs.org.au
CT-F-129v13 15/12/21

1. PATIENT DETAILS

LAST NAME GIVEN NAME | DATE OF BIRTH | LABORATORY REF

ADDRESS POST CODE | PHONE (HOME) | **MOBILE**

2. TEST REQUESTED

percept non-invasive prenatal test

Tests for whole chromosome changes of all 23 chromosome pairs (inclusive of 21, 18, 13, X and Y) and copy number changes ≥ 7 Mb for chromosomes 1-22.

3. CLINICAL INFORMATION

☐ SINGLETON ☐ TWIN ☐ TRIPLET

GESTATIONAL AGE: ___________________ as of date: _____________

Gestation must be at least 10 weeks at collection; 12 weeks for triplets

EDD: (dd/mm/yyyy): ________________________________

MATERNAL WEIGHT (kg): _____________________________

MATERNAL HEIGHT (cm): _____________________________

4. TEST INDICATIONS

☐ *percept* AS PRIMARY SCREENING TEST

☐ COMBINED 1ST TRIMESTER SCREENING RESULT

T21: 1/________ T18: 1/________ T13: 1/________

☐ ULTRASOUND ABNORMALITY: _______________________

☐ KNOWN TRANSLOCATION CARRIER (prior lab assessment required)

Specify: ____

☐ OTHER: ____

I verify that the patient & prescriber information in this form is complete & accurate to the best of my knowledge.

DOCTOR'S SIGNATURE AND REQUEST DATE

SIGNATURE: DATE:

COPY REPORTS TO:

5. REQUESTING DOCTOR (PROVIDER #, INITIALS, ADDRESS, EMAIL)

Email:

NIPT-08

6. PATIENT CONSENT

By signing this form, I request that VCGS perform the *percept* prenatal test. I have read the patient consent included on the back of this form. The risks & limitations of this test have been adequately explained to me.

PATIENT SIGNATURE AND DATE

SIGNATURE: DATE:

NOTES:

This test is validated for pregnancies of at least 10 weeks gestational age.
Fetal sex is always reported. Clinician to disclose to patient on request.
Sex chromosome aneuploidy cannot be detected in twins/triplets.

PHLEBOTOMIST DETAILS:

Time of collection:

Date of collection:

SIGNATURE:

Place of collection:

7. PATIENT PAYMENT INFORMATION

PLEASE NOTE: **NIPT IS NOT COVERED BY MEDICARE OR PRIVATE HEALTH INSURANCE**

Please make sure your mobile number is provided in the patient details section above.
You will receive an SMS with a link for payment once your sample has been received.

Your results will be sent to your doctor once testing is complete and payment has been made.
This is usually within 3-5 days after your sample has been received by VCGS.

VCGS Accounts for enquiries:
P 1300 557 779

VCGS
leaders in genetic health

Victorian Clinical Genetics Services
The Royal Children's Hospital
Flemington Road, Parkville VIC 3052
P +61 1300 118 247 **F** +61 3 8341 6366
W vcgs.org.au

Patient Consent

Genetic counselling with a VCGS genetic counsellor (03 9936 6402) is available to anybody considering this screening test. VCGS genetic counsellors can also discuss other prenatal screening and testing options with you. More information about this test and the chromosome conditions included in the test is available at vcgs.org.au/perceptNIPT

Patients having *percept* non-invasive prenatal testing should be aware of the following key points:

Purpose of the test

This test identifies pregnancies at 'high risk' of:

- The common trisomies 21 (Down syndrome), 18 (Edwards syndrome) and 13 (Patau syndrome);
- Conditions caused by too many or too few of the sex chromosomes (X and Y);
- Rare autosomal trisomies (those involving chromosomes other than 21, 18, 13, X and Y);
- Extra or missing copies of large parts of chromosomes (≥7Mb) that are known to be associated with health concerns.

Test process

- This test is intended to be performed from the 10th week of pregnancy onwards, as determined by a dating ultrasound.
- A sample of your blood will be collected and sent to VCGS who will issue a report to your healthcare provider. Your healthcare provider is responsible for interpreting and explaining your test results. VCGS genetic counsellors are also available to discuss your results with you.
- The test results will include the sex of the pregnancy. If you do not wish to know the sex you can ask your healthcare provider not to disclose it to you. However, if the results show too many or too few of the sex chromosomes, you may not be able to avoid learning the sex of your pregnancy.
- As this is a screening test, it is recommended that all high risk test results are confirmed by chromosome analysis through CVS or amniocentesis.

Limitations of the test

- This test screens for extra or missing copies of whole chromosomes in the fetus and is not designed to detect small genetic imbalances, single gene disorders or non-genetic causes of fetal abnormalities. Missing or extra parts of the chromosomes 1-22 (≥7Mb in size) are reported when they are known to be associated with health concerns.
- Low risk test results do not eliminate the possibility that your pregnancy may have other chromosomal abnormalities, birth defects, or other conditions, such as open neural tube defects. A 'low risk' result does not guarantee a healthy pregnancy or baby.
- As this is a screening test, there is a small possibility that the results could be incorrect. It is possible to receive a high risk result even though a chromosomal abnormality is not present in the fetus. This is called a 'false positive' result. It is also possible that the chromosomal abnormality being tested for could be present even if the result is low risk. This is called a 'false negative' result.
- Some high risk test results may be due to chromosomal changes in the mother. Further testing of the mother may be required in some circumstances.
- The ability of this test to accurately report fetal sex chromosome abnormalities (too many or too few sex chromosomes) is not well known. Incorrect test results may occur more frequently for these conditions.
- For technical and biological reasons, the fetal sex is reported with >99% accuracy (not 100%).
- *percept* NIPT may be used to screen a pregnancy where one parent is a known translocation carrier. This testing is only performed by prior arrangement.
- The ability of this test to detect chromosome abnormalities in a triplet pregnancy is not well known. Incorrect test results may occur more frequently.

Privacy, confidentiality and use of information

- Your test results will be kept confidential. Results will only be released to your healthcare provider, other healthcare providers involved in your medical care, or to another healthcare provider as directed by you, or otherwise as required or authorised by applicable law.
- Collecting information on your pregnancy after testing is part of our laboratory's standard practice for quality purposes and test evaluation. VCGS may contact your healthcare provider to obtain this information.
- To advance scientific knowledge, de-identified genetic and health information may be included in scientific presentations, publications and education resources for health care providers.

Retention and use of samples

- In line with best practices and clinical laboratory standards, leftover de-identified specimens (unless prohibited by law), de-identified genetic material, as well as other information learned from your testing, may be used by VCGS for purposes of quality control, laboratory operations, laboratory test development, laboratory improvement, and generation of new scientific knowledge. All such uses will be in compliance with applicable law.

Financial responsibility statement

- You are responsible for fees incurred with VCGS for services performed.

NIPT-08
