# Supplementary material for: The Function of Non-invasive Prenatal Testing (NIPT) Request Forms in the Australian Context
Source: Health Care Anal. 2025 Oct 24;34(3):783–807. doi: 10.1007/s10728-025-00543-0 (PMC13675290; doi:10.1007/s10728-025-00543-0)
Supplement: Supplementary file 2 [file 10728_2025_543_MOESM2_ESM.pdf]

The Function of Non-invasive Prenatal Testing (NIPT) Request Forms in the Australian Context

Supplementary material – list of forms

Form identifier	Brand or name of test	Name of Australian provider and/or service	Date of document	Date ascertained
**NIPT-01**	Generation	Genomic Diagnostics; Healius Pathology	May 2023	Internal date stamp
**NIPT-02**	Harmony	Australian Clinical Labs; Roche	April 2023	Internal date stamp
**NIPT-03**	N/A*	Sonic Genetics	May 2023	Internal date stamp
**NIPT-04**	Nest	Repromed (Monash IVF)	April 2023	File name: *Editable-SA-nest-pathology-request-form-v2023-APR-2 [.pdf]*
**NIPT-05**	Nest+**	Repromed (Monash IVF)	June 2023	File name: *MI2342-Nest-Prenatal-Screening-Request-Form-Singleton-V3-05JUN23 [.pdf]*
**NIPT-06**	NIFTY	Genomics For Life	October 2023	Internal date stamp
**NIPT-07**	Panorama	Virtus Diagnostics	June 2020	Internal date stamp
**NIPT-08**	percept	Victorian Clinical Genetics Services	December 2021	Internal date stamp

*Sonic Genetics offered the Harmony test from 2015, and from 2020 also used the VeriSeq NIPT Solution v2 assay. They ceased use of the Harmony test in December 2021. See: https://www.sonicgenetics.com.au/patient/test-information/nipt/nipt-faqs/ (Accessed 23 July 2025).

**Date taken from original file name of download available on the Nest website; internal date stamp indicates 2021, but it also differs from the older 2021 versions of the form.
